# Pedal peptide/orcokinin‐type neuropeptide signaling in a deuterostome: The anatomy and pharmacology of starfish myorelaxant peptide in *Asterias rubens*

**DOI:** 10.1002/cne.24309

**Published:** 2017-09-06

**Authors:** Ming Lin, Michaela Egertová, Cleidiane G. Zampronio, Alexandra M. Jones, Maurice R. Elphick

**Affiliations:** ^1^ Queen Mary University of London, School of Biological & Chemical Sciences, Mile End Road London UK; ^2^ School of Life Sciences and Proteomics Research Technology Platform University of Warwick Coventry UK

**Keywords:** deuterostome, echinoderm, neuropeptide, orcokinin, pedal peptide, starfish myorelaxant peptide

## Abstract

Pedal peptide (PP) and orcokinin (OK) are related neuropeptides that were discovered in protostomian invertebrates (mollusks, arthropods). However, analysis of genome/transcriptome sequence data has revealed that PP/OK‐type neuropeptides also occur in a deuterostomian phylum—the echinoderms. Furthermore, a PP/OK‐type neuropeptide (starfish myorelaxant peptide, SMP) was recently identified as a muscle relaxant in the starfish *Patiria pectinifera*. Here mass spectrometry was used to identify five neuropeptides (ArPPLN1a‐e) derived from the SMP precursor (PP‐like neuropeptide precursor 1; ArPPLNP1) in the starfish *Asterias rubens*. Analysis of the expression of ArPPLNP1 and neuropeptides derived from this precursor in *A. rubens* using mRNA in situ hybridization and immunohistochemistry revealed a widespread pattern of expression, with labeled cells and/or processes present in the radial nerve cords, circumoral nerve ring, digestive system (e.g., cardiac stomach) and body wall‐associated muscles (e.g., apical muscle) and appendages (e.g., tube feet and papulae). Furthermore, our data provide the first evidence that neuropeptides are present in the lateral motor nerves and in nerve processes innervating interossicular muscles. In vitro pharmacological tests with SMP (ArPPLN1b) revealed that it causes dose‐dependent relaxation of apical muscle, tube foot and cardiac stomach preparations from *A. rubens*. Collectively, these anatomical and pharmacological data indicate that neuropeptides derived from ArPPLNP1 act as inhibitory neuromuscular transmitters in starfish, which contrasts with the myoexcitatory actions of PP/OK‐type neuropeptides in protostomian invertebrates. Thus, the divergence of deuterostomes and protostomes may have been accompanied by an inhibitory–excitatory transition in the roles of PP/OK‐type neuropeptides as regulators of muscle activity.

## 1 INTRODUCTION

Neuropeptides are evolutionarily ancient neuronal signaling molecules that regulate diverse physiological processes and behaviors in animals. The isolation and structural identification of neuropeptides was first accomplished through use of bioassays to monitor chromatographic purification of pharmacologically active components in neural extracts (O'Shea & Schaffer, 1985). For example, using this approach the cardioactive neuropeptide FMRFamide was discovered in bivalve mollusks (Price & Greenberg, 1977). A different approach to neuropeptide discovery was adopted by Lloyd and Connolly (1989)—they sought to identify neuropeptides in the mollusk *Aplysia californica* that are synthesized preferentially in particular ganglia. A neuropeptide that is synthesized by cell bodies in the pedal ganglia was purified and sequenced (PLDSVYGTHGMSGFA) and then appropriately named “pedal peptide” (PP; Lloyd & Connolly, 1989).

Immunohistochemical localization of PP in *Aplysia* revealed that it is synthesized by a population of neurons located mostly in the pedal ganglia with processes projecting peripherally and predominantly innervating the foot (Hall & Lloyd, 1990; Pearson & Lloyd, 1989). Consistent with this pattern of expression, PP causes an increase in the amplitude and relaxation rate of nerve‐evoked contractions of *Aplysia* foot muscle (Hall & Lloyd, 1990). Furthermore, PP‐expressing neurons fire in phase with each pedal wave during locomotor activity and stop firing during defensive contractions of the foot (Hall & Lloyd, 1990). Thus, PP‐releasing neurons appear to modulate foot muscle contractility during locomotion. However, PP is not only involved in control of foot activity because PP‐expressing neurons also innervate other organs (e.g., the hermaphroditic duct) (Hall & Lloyd, 1990). Furthermore, sequencing of the *Aplysia* neural transcriptome revealed that PP is just one of a large family of related neuropeptides in this species, which are derived from four precursor proteins (Moroz et al., 2006).

Multiple PP‐type neuropeptides and precursor proteins have also been identified in other mollusks, including the limpet *Lottia gigantea* (Veenstra, 2010) and the nudibranch *Tritonia diomedia* (Lloyd, Phares, Phillips, & Willows, 1996). Investigation of the pharmacological effects of PPs in *Tritonia* revealed that they cause an increase in the ciliary beat frequency of cells in the pedal epithelium, again providing evidence of a physiological role in regulation of locomotor activity (Willows, Pavlova, & Phillips, 1997). Furthermore, immunohistochemical analysis of PP expression in *Tritonia* indicated roles in a variety of other behavioral activities, including orientation, swimming, and feeding (Beck, Cooper, & Willows, 2000; Gaston, 1998). Accordingly, a PP was found to cause an increase in the ciliary beat frequency of cells in the salivary duct of *Tritonia*, indicative of a physiological role in regulation of salivary transport associated with feeding (Gaston, 1998).

Molluscan PP‐type neuropeptides belong to a family of related neuropeptides that also occur in other phyla, including arthropod orcokinins (Jekely, 2013; Rowe & Elphick, 2012). Orcokinin was first identified in the crayfish *Orconectes limosus* as a neuropeptide (NFDEIDRSGFGFN) that is a potent stimulator of the hind‐gut, enhancing the frequency and amplitude of spontaneous contractions (Stangier, Hilbich, Burdzik, & Keller, 1992). Molecular characterization of orcokinins in the crayfish *Procambarus clarkia* revealed that orcokinin and other orcokinin‐like peptides are derived from two closely related precursor proteins—preproorcokinin‐A and ‐B (Yasuda‐Kamatani & Yasuda, 2000). Furthermore, orcokinin‐type neuropeptides have also been identified in other arthropods, including insects. This has revealed that multiple orcokinin isoforms occur in each species (Pascual, Castresana, Valero, Andreu, & Belles, 2004) and, as in crustaceans, alternatively spliced transcripts encoding preproorcokinin‐A and ‐B have been identified in *Drosophila melanogaster* and other insects (Liu et al., 2006; Sterkel et al., 2012; Veenstra & Ida, 2014).

Immunohistochemical investigation of the expression of orcokinins in the crayfish *Orconectes limosus* revealed immunostained cell bodies in the sixth abdominal ganglion with axonal processes that innervate hind‐gut muscles, consistent with the myoexcitatory effects of orcokinins on the hind‐gut (Dircksen, Burdzik, Sauter, & Keller, 2000). Orcokinin‐expressing neurons have also been mapped in the stomatogastric nervous system of three crustacean species and accordingly orcokinins alter the rhythmic motor output of the stomatogastric nervous system (Li et al., 2002; Skiebe, Dreger, Meseke, Evers, & Hucho, 2002). Thus, orcokinins act as neuromodulators in the central nervous system and the peripheral neuromuscular system of crustaceans.

Immunohistochemical localization of orcokinins in insects has revealed widespread patterns of expression in the brain (Hofer, Dircksen, Tollback, & Homberg, 2005). More specifically, the presence of orcokinin‐expressing neurons in a region of the optic lobes known as the accessory medulla was noted because this region of the insect brain houses the master circadian clock that controls circadian locomotor activity. Accordingly, injection of an orcokinin into the accessory medulla of cockroaches causes a phase‐dependent shift in circadian locomotor activity (Hofer & Homberg, 2006). More recent studies on orcokinin function, employing use of RNA interference‐based gene knockdown methods, indicate that orcokinins are regulators of “awakening” behavior in the beetle *Tribolium castaneum* (Jiang, Kim, & Park, 2015) and regulators of ecdysis in the kissing bug *Rhodnius prolixus* (Wulff et al., 2017).

Genome/transcriptome sequencing has enabled the discovery of PP/orcokinin (PP/OK)‐type neuropeptides in other phyla, aside from mollusks and arthropods. Thus, two precursors of multiple PP‐like neuropeptides were identified in the annelid *Capitella teleta* (Veenstra, 2011) and a precursor of orcokinin‐like peptides was identified in the tardigrade *Milnesium tardigradum* (Christie et al., 2011). Furthermore, discovery of two genes encoding PP/OK‐type precursors in an echinoderm species, the sea urchin *Strongylocentrotus purpuratus*, provided new insights on the evolution of this neuropeptide family (Rowe & Elphick, 2012). These were the first genes encoding PP/OK‐type neuropeptides to be discovered in a deuterostome, which was an important finding because it revealed that the evolutionary history of PP/OK‐type neuropeptides can be traced back to the common ancestor of protostomes and deuterostomes. Thus far, genes encoding PP/OK‐type neuropeptides have not been identified in other deuterostomian invertebrates (i.e., hemichordates, cephalochordates, urochordates) or vertebrates. Therefore, it is possible that within the deuterostomian branch of the animal kingdom PP/OK‐type neuropeptides have been retained only in the echinoderm lineage. Interestingly, it was the discovery of PP/OK‐type neuropeptides in *S. purpuratus* that first enabled recognition that molluscan PPs and arthropodan orcokinins are members of a same bilaterian neuropeptide family (Rowe & Elphick, 2012). Hitherto, research on PPs in mollusks and orcokinins in arthropods had been pursued independently without reported recognition that these neuropeptides are related. Furthermore, identification of the PP/OK‐type neuropeptide precursors in *S. purpuratus* also facilitated identification of two neuropeptide precursors (NPL14, NPL15) in the nematode *Caenorhabditis elegans* (*C. elegans* Sequencing Consortium, 1998) as precursors of PP/OK‐type neuropeptides. Thus, neuropeptides from lophotrochozoan protostomes (mollusks, annelids), ecdysozoan protostomes (arthropods, nematodes) and deuterostomes (echinoderms) were unified for the first time as members of a bilaterian family of PP/OK‐type peptides (Rowe & Elphick, 2012). Subsequently, the relationship between PPs and orcokinins was demonstrated independently using similarity‐based clustering methods (Jekely, 2013).

The discovery of two PP/OK‐type neuropeptide precursors (SpPPLNP1 and SpPPLNP2) in an echinoderm, the sea urchin *S. purpuratus*, has provided a basis for investigation of the physiological roles of PP/OK‐type neuropeptides in the deuterostomian branch of the animal kingdom. The structures of PP/OK‐type neuropeptides derived from SpPPLNP1 and SpPPLNP2 have been determined by mass spectroscopic analysis of extracts of *S. purpuratus* (Menschaert et al., 2010; Rowe & Elphick, 2012). Furthermore, one of the peptides derived from SpPPLNP1 (SpPPLN1c) has been tested for myoactivity on sea urchin tube foot and esophagus preparations, but no effects were observed (Rowe & Elphick, 2012).

A major advance in our knowledge of PP/OK‐type neuropeptide function in echinoderms was accomplished recently with the discovery that a myorelaxant purified from the starfish species *Patiria pectinifera* is a member of the PP/OK‐type neuropeptide family (Kim et al., 2016). Using the apical muscle of *P. pectinifera* as a bioassay for myoactive peptides, a peptide that causes relaxation of the apical muscle was purified and sequenced (FGKGGAYDPLSAGFTD) and named starfish myorelaxant peptide (SMP). Cloning and sequencing of a cDNA encoding the *P. pectinifera* SMP precursor revealed that it comprises 12 copies of SMP and 3 related peptides (7 copies in total). Furthermore, analysis of the sequence of SMP and the SMP precursor revealed that SMP is a PP/OK‐type neuropeptide (Kim et al., 2016). Investigation of the expression of the SMP precursor in *P. pectinifera* using quantitative PCR revealed that it is widely expressed in this starfish species, with the highest levels of expression in the radial nerve cords and lower levels of expression in the apical muscle, tube feet, coelomic lining, cardiac stomach, pyloric stomach, and pyloric caeca (Kim et al., 2016). Consistent with this widespread pattern of expression it was found that SMP, in addition to causing relaxation of the apical muscle, also causes relaxation of cardiac stomach and tube foot preparations from *P. pectinifera*. Further insights into the physiological roles of SMP could be obtained by investigating its anatomical pattern of expression in starfish.

A homolog of the *P. pectinifera* SMP precursor has been identified in another starfish species—the common European starfish *Asterias rubens* (Kim et al., 2016). We also refer to the *A. rubens* SMP precursor as *A. rubens* PP‐like neuropeptide precursor 1 (or ArPPLNP1) because a partial sequence of a second PP‐type precursor has also been identified in *A. rubens* (Semmens et al., 2016). This second precursor was originally named ArPPLNP (Semmens et al., 2016) but henceforth we will refer to it as ArPPLNP2 to distinguish it from the SMP‐type precursor ArPPLNP1.

*Asterias rubens* is an established model echinoderm for research on neuropeptide signaling. For example, the first anatomical investigation of neuropeptide expression in starfish reported the distribution of FMRFamide‐like immunoreactivity in *A. rubens* (Elphick, Emson, & Thorndyke, 1989). Subsequently, the first echinoderm neuropeptides to be sequenced, the SALMFamides S1 and S2, were purified from *A. rubens* (Elphick, Price, Lee, & Thorndyke, 1991). Immunohistochemical analysis of S1 and S2 expression revealed that both peptides are widely expressed in *A. rubens* (Newman, Elphick, & Thorndyke, 1995a, 1995b) and in vitro pharmacological experiments revealed that both S1 and S2 act as muscle relaxants in starfish (Elphick, Newman, & Thorndyke, 1995; Melarange, Potton, Thorndyke, & Elphick, 1999). More recently, transcriptome sequencing has enabled identification of forty neuropeptide precursors in *A. rubens* (Semmens et al., 2016). Furthermore, functional characterization of neuropeptides derived from these precursors is being facilitated by use of mass spectrometry to determine peptide structure (Lin et al., 2017; Semmens et al., 2013), mRNA in situ hybridization to analyze patterns of neuropeptide precursor expression (Lin et al., 2017) and in vitro pharmacological assays to determine neuropeptide actions (Lin et al., 2017; Semmens et al., 2013).

Here we have used mRNA in situ hybridization and immunohistochemistry (employing use of novel antibodies) to investigate the expression patterns of ArPPLNP1 and neuropeptides derived from this precursor in *A. rubens*. This is the first study to examine the expression of PP/OK‐type neuropeptides at the cellular level in a deuterostome. Informed by the anatomical data obtained, the in vitro pharmacological actions of one of the neuropeptides derived from ArPPLNP1 (ArSMP or ArPPLN1b) were also examined. Collectively, our findings provide new insights into the physiological roles of PP/OK‐type neuropeptides in starfish.

## 2 MATERIALS AND METHODS

### 2.1 Animals

Starfish (*A. rubens*) with a diameter > 4 cm were collected at low tide from the Thanet coast (Kent, UK) or were obtained from a fisherman based at Whitstable (Kent, UK). These animals were maintained in a circulating seawater aquarium at ∼12°C in the School of Biological and Chemical Sciences at Queen Mary University of London and were fed on mussels (*Mytilus edulis*). Smaller juvenile specimens of *A. rubens* (diameter 0.5–1.5 cm) were collected at the University of Gothenberg Sven Lovén Centre for Marine Infrastructure (Kristineberg, Sweden) and were fixed in Bouin's solution.

### 2.2 Mass spectrometric identification of peptides derived from ArPPLNP1 in extracts of *A. rubens* radial nerves

Mass spectrometry was used to determine the structures of neuropeptides derived from ArPPLNP1 by analysis of *A. rubens* radial nerve cords extracted in 90% methanol/9% acetic acid and then reduced and alkylated, as described previously (Lin et al., 2017). As the methods employed here have been described in detail previously (Lin et al., 2017), here only a short overview of the methods is reported. The pH of the radial nerve cord extract was adjusted using ammonium bicarbonate, and the extract was reduced with dithiothreitol (DTT) and alkylated using iodoacetamide but not treated with trypsin or any other proteolytic enzyme. Samples of the extract were analysed by nanoLC‐ESI‐MS/MS using an Orbitrap Fusion (ThermoScientific) and data analysis was performed as described in Lin et al. (2017), except that more recent versions of Mascot (Matrix Science, London, UK; version 2.5.0) and Scaffold (version Scaffold_4.5.3, Proteome Software Inc., Portland, OR) were used to annotate spectra. Mascot was searched with a fragment ion mass tolerance of 0.050 Da and a parent ion tolerance of 10.0 PPM. Conversion of glutamine to pyroglutamate at the N‐terminus, amidation of the C‐terminus, oxidation of methionine residues and carbamidomethyl of cysteine residues were specified in Mascot as variable modifications.

### 2.3 Localization of ArPPLNP1 expression in *A. rubens* using mRNA in situ hybridization

A cDNA encoding the complete open read frame (ORF) of ArPPLNP1 (GenBank accession number: KT870153) has been cloned and sequenced, as described previously (Kim et al., 2016). Prior to this, a cDNA encoding a partial ORF (477 bases) was cloned by PCR using the following forward and reverse primers, respectively: GCTTCACAGACAAGCGTTT and TACACACCAAGCAGTGACA. A pBluescript SKII (+) vector containing the 477 base cDNA was then used as a template for production of digoxygenin (DIG)‐labeled RNA probes.

The methods employed for (a) production of antisense and sense DIG‐labeled RNA probes, (b) preparation of paraffin and frozen sections of fixed specimens of *A. rubens* and (c) visualization of ArPPLNP1 transcripts in sections of *A. rubens* were the same as those reported recently for the *A. rubens* relaxin‐like gonad‐stimulating peptide precursor (AruRGPP) (Lin et al., 2017).

### 2.4 Production and characterization of rabbit antibodies to ArPPLNP1‐derived neuropeptides

As ArPPLN1b (ArSMP) is the most abundant of the peptides derived from ArPPLNP1 (four out of nine peptide copies) it was selected as an antigen for generation of antibodies to be used for immunhistochemical localization of ArPPLN1‐type peptides in *A. rubens*. A peptide comprising the C‐terminal twelve amino acid residues of ArPPLN1b with the addition of an N‐terminal lysine residue (KGAFDPLSAGFTD) was designed as an antigen (ag) peptide (ArPPLN1b‐ag) and synthesized by Peptide Protein Research Ltd. (Hampshire, UK). ArPPLN1b‐ag was conjugated to porcine thyroglobulin (Sigma‐Aldrich) as a carrier protein, using 5% glutaraldehyde in phosphate buffer (PB, 0.1 M, pH 7.2) as a coupling reagent. Following dialysis in distilled water to remove glutaraldehyde and any uncoupled peptide, the conjugate solution was divided into aliquots containing approximately 50 nmol conjugated antigen peptide per tube and then frozen. A rabbit was used for antibody production with immunization and serum collection performed by Charles River Labs (Margate, UK) according to the following protocol. On day 0 pre‐immune serum was collected and the first immunization (∼100 nmol of conjugated ArPPLN1b‐ag in Freund's complete adjuvant) was administered. Booster immunizations (∼50 nmol of conjugated ArPPLN1b‐ag in Freund's incomplete adjuvant) were administered on days 28, 42, and 56). Antiserum samples were collected on days 37 and 51 and a final bleed was collected on day 70.

To assess production of antibodies during the immunization protocol and following collection of a terminal bleed, antisera were tested for antibodies to ArPPLN1b‐ag using Enzyme‐Linked ImmunoSorbent Assays (ELISA) in two formats. Firstly, an assay where a fixed amount of ArPPLN1b‐ag peptide (100 µL of 1 µM) was added to each well and then incubated with varying dilutions of pre‐immune serum or antiserum (10^−3^–10^−8^ diluted in 5% goat serum/PBS; *n* = 2). Secondly, an assay where varying amounts of ArPPLN1b‐ag peptide (100 µL; 10^−6^–10^−11^ M; *n* = 2) was added to each well and then incubated with pre‐immune serum or antiserum at a fixed dilution of 10^−4^. For both assay formats the ArPPLN1b‐ag peptide was dissolved in carbonate/bicarbonate buffer (25 mM anhydrous sodium carbonate, 25 mM sodium bicarbonate, pH 9.8) and added to wells of a polystyrene microtiter plate (Microlon**^®^**; Greiner Bio‐One International), which was then covered with Parafilm M^®^ (Starlab) and incubated overnight at 4°C. The following day the liquid contents of the plate were disposed of, the wells were rinsed with 200 μl PBS (3 × 10 min) and the plate was drained on blotting paper. Then 200 µL of a blocking solution containing 5% goat antiserum/PBS was added to each well and left at room temperature for 2 hr. The blocking buffer was discarded and each well was washed with PBS containing 0.1% Tween20 (PBST; 200 μl, 3 × 10 min) and then the plate was drained, as above. Antiserum diluted in 5% goat antiserum (Sigma‐Aldrich)/PBS was added to each well and incubated overnight at 4°C. The antiserum was discarded and each well was washed with PBST (200 μl; 3 × 10 min) and then the plate was drained. Alkaline phosphatase‐conjugated goat anti‐rabbit IgG secondary antibodies (Vector Laboratories; diluted 1:3,000 in 5% normal goat serum/PBST) were added to each well and incubated for 3 hr at room temperature. After washing the plate with PBST (4 × 10 min), 100 μl *p*‐Nitrophenylphosphate Alkaline Phosphatase Substrate (pNPP, Vector Laboratories) prepared in carbonate/bicarbonate buffer was added to each well and after a 20 min incubation at room temperature the absorbance at 415 nm was measured using FLUOstar Omega (BMG LABTECH ‐ The Microplate Reader Company). Mean absorbance values were calculated and plotted using Prism 6.0c.

### 2.5 Localization of ArPPLN1b in *A. rubens* using immunohistochemistry

For immunohistochemistry, small specimens of *A. rubens* (< 6 cm diameter) were fixed in Bouin's solution (75 mL saturated picric acid in seawater, 25 mL 37% formaldehyde, 5 mL acetic acid) at 4°C for 2 days. Fixed starfish were decalcified in 2% ascorbic acid/0.15 M sodium chloride (∼ 1 week at 4°C with regular changes of the solution), embedded in paraffin wax, sectioned (8–12 µm) using a Leica RM2145 microtome and mounted on chrome alum‐gelatin coated glass slides.

Xylene was used to remove wax from sections (3 × 10 min at room temperature) and then slides were incubated in 100% ethanol (2 × 10 min). The slides were incubated in 1% hydrogen peroxide in methanol for 30 min to quench endogenous peroxidase and then rehydrated through a graded series of ethanol (90%, 70%, 50%; 10 min for each) into distilled water. Subsequent steps were the same as those described previously for immunohistochemistry with 1E11 monoclonal antibodies (Lin et al., 2017), with the exception of the primary antibodies (rabbit ArPPLN1b antiserum diluted to 10^−5^ in 5% goat serum/PBST) and secondary antibodies (goat anti‐rabbit horseradish peroxidase conjugated immunoglobulins [Jackson ImmunoResearch via Stratech Scientific, Newmarket, Suffolk, UK] diluted 1:500 or 1:1,000 in 2% goat serum/PBST) used. Immunostaining was visualised using diaminobenzidine (DAB, VWR Chemicals) and when intense immunostaining was observed, the DAB was washed off with distilled water (10 min). Following dehydration through an ethanol series (50%, 70%, 90%, 2 × 100%; 10 min each), slides were cleared in xylene (2 × 10 min) and mounted with coverslips using DPX Mountant (VWR Chemicals).

To assess the specificity of immunostaining, control experiments were performed where slides were incubated with antiserum pre‐absorbed with the antigen peptide. Antiserum pre‐absorption was performed by incubating the antiserum diluted to 10^−4^ in PBS with ArPPLN1b‐ag (200 µM) for 1–2 hr on a rocking shaker at room temperature. Then the pre‐absorbed antiserum was diluted ten fold to 10^−5^ in 5% goat antiserum/PBST and tested on starfish sections, as described above.

To facilitate interpretation of immunostaining in some regions of the starfish body, sections adjacent to immunostained sections were stained using Masson's trichrome staining, which differentiates collagenous (blue) from non‐collagenous (red) tissue. The method employed for trichrome staining of starfish sections has been described recently (Blowes et al., 2017).

Photographs of immunostained and trichrome stained sections were obtained and assembled into montages, as described previously (Lin et al., 2017). A QIClick CCD Camera (QImaging) linked to a DMRA2 light microscope (Leica) and Volocity v.6.3.1 image analysis software (Perkin‐Elmer, Boston, MA) running on an iMac computer (27‐inch with OS X Yosemite, v. 10.10) were used to capture the photographs. Images were compiled into montages and labeled using Photoshop CC (2015.0.0; Adobe Systems, San Jose, CA), including use of cropping and contrast adjustment tools, on a MacBook Pro computer (13‐inch, with OS X EI Capitan).

### 2.6 In vitro pharmacology

**Figure 1:**
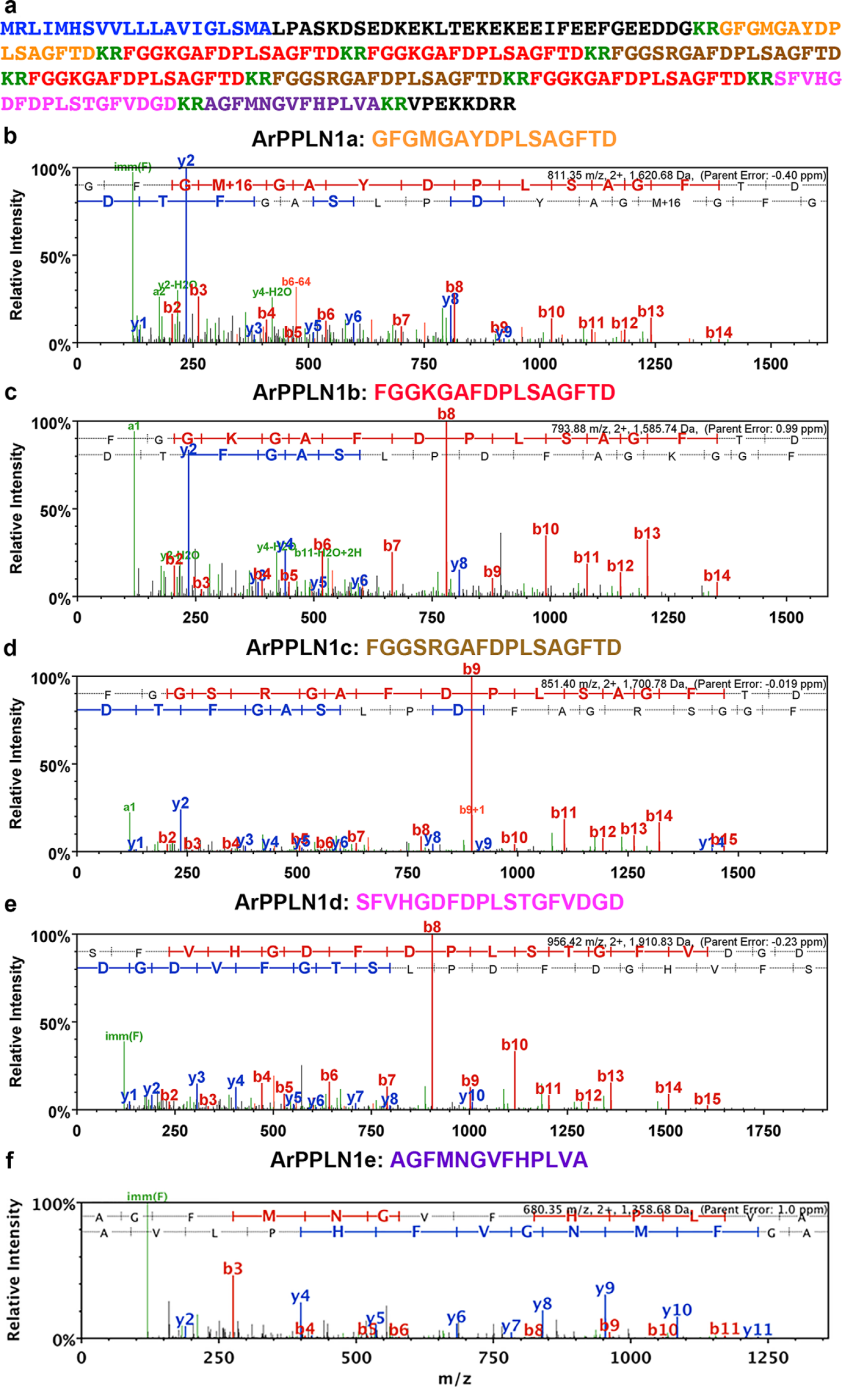
Mass spectrometric identification of peptides derived from ArPPLNP1 in an extract of *A. rubens* radial nerve cords. (a) Amino acid sequence of ArPPLNP1 with the predicted signal peptide shown in blue, predicted cleavage sites shown in green and peptides derived from the precursor shown in orange (ArPPLN1a), red (ArPPLN1b), brown (ArPPLN1c), pink (ArPPLN1d) and purple (ArPPLN1e). (b–f) annotated MS/MS spectra for ArPPLN1a‐e, respectively. The b series of peptide fragment ions are shown in red, the y series in blue and additional identified peptide fragment ions in green. The amino acid sequence identified in the mass spectrum is highlighted at the top of each panel and M + 16 represents oxidized methionine. (b) MS/MS spectrum for the ArPPLN1a (GFGMGAYDPLSAGFTD) observed at 811.35 *m/z*, 2+ ion, with precursor mass error −0.40 ppm (Mascot score 84). (c) MS/MS spectrum for ArPPLN1b (FGGKGAFDPLSAGFTD) observed at 793.88 *m/z*, 2+ ion, with precursor mass error 0.99 ppm (Mascot score 61). (d) MS/MS spectrum for ArPPLN1c (FGGSRGAFDPLSAGFTD) observed at 851.40 *m/z*, 2+ ion, with precursor mass error −0.019 ppm (Mascot score 60). (e) MS/MS spectrum for ArPPLN1d (SFVHGDFDPLSTGFVDGD) observed at 956.42 *m/z*, 2+ ion, with precursor mass error −0.23 ppm (Mascot score 69). (f) MS/MS spectrum for ArPPLN1e (AGFMNGVFHPLVA) observed at 680.35 *m/z*, 2+ ion, with precursor mass error 1.0 ppm (Mascot score 42)

ArPPLN1b (ArSMP) was selected for in vitro pharmacological tests because it is the most abundant (four copies) of the five neuropeptides derived from ArPPLNP1 (Figure 1a). The structure of this peptide having been confirmed by mass spectrometry (Figure 1c), it was synthesized commercially by Peptide Protein Research Ltd. (Hampshire, UK). ArPPLN1b was tested on apical muscle, tube foot and cardiac stomach preparations from *A. rubens*, with the SALMFamide neuropeptide S2 (SGPYSFNSGLTF‐NH_2_) tested as a control peptide that causes relaxation of all three of these preparations (Elphick et al., 1995; Kim et al., 2016; Melarange & Elphick, 2003).

Apical muscles were dissected from the aboral body wall of starfish arms and cut into segments approximately 1 cm in length (Melarange & Elphick, 2003) and cotton ligatures were tied at each end. Tube foot preparations were obtained by dissecting from starfish arms a small square‐shaped piece of ambulacral body wall containing a single intact tube foot stem and its associated ampulla. Cotton ligatures were tied around the body wall and the tube foot sucker, as illustrated previously (Melarange & Elphick, 2003). Cardiac stomach preparations were dissected as described and illustrated previously (Elphick et al., 1995) and then cotton ligatures were tied around the esophagus and around the aboral region of the cardiac stomach.

Ligatures at one end of the preparation (body wall segment and esophagus for tube foot and cardiac stomach preparations, respectively) were attached to a fixed metal hook in a 20 mL glass organ bath containing artificial seawater at ∼11°C. The other ligature was tied to a High Grade Isotonic Transducer (ADinstruments MLT0015) connected to PowerLab data acquisition hardware (ADinstruments PowerLab 2/26). Output from Powerlab was recorded using LabChart (v8.0.7) software installed on a laptop computer (Lenovo E540, Windows 7 Professional).

The resting tension applied to preparations was adjusted to 0.25 g followed by an equilibration period in artificial seawater (ASW) of ∼ 20 min. Then preparations were induced to contract by application of 10 µM acetylcholine (ACh; for apical muscle and tube foot preparations) or ASW containing 30 mM added KCl (cardiac stomach preparations). Once a stable baseline contracted state was reached, synthetic ArPPLN1b or S2 was added to sequentially achieve organ bath concentrations between 10^−10^ M and 10^−5^ M. Cumulative dose‐response curves were constructed by expressing relaxation as a percentage reversal of the contraction induced by ACh or KCl. Each peptide concentration was tested on at least five preparations.

### 2.7 In vivo pharmacology

Previous studies have revealed that an ArPPLN1b‐like peptide (SMP) causes dose‐dependent relaxation of in vitro preparations of the cardiac stomach from *P. pectinifera* (Kim et al., 2016). The SALMFamide neuropeptide S2 also causes dose‐dependent cardiac stomach relaxation in *A. rubens* (Elphick et al., 1995; Melarange et al., 1999) and in *P. pectinifera* (Kim et al., 2016). Furthermore, consistent with the in vitro actions of S2, in vivo injection of S2 into *A. rubens* triggers eversion of the cardiac stomach (Melarange et al., 1999). Therefore, here we investigated if injection of ArPPLN1b also triggers cardiac stomach eversion in *A. rubens*, employing the same method as reported previously for tests with S2 (Melarange et al., 1999). Specimens of *A. rubens* (6–12 cm in diameter) were starved for three days and then each starfish was moved one at a time to glass testing tank. Ten starfish were injected with 100 µL of 1 mM ArPPLN1b at two or three sites in the aboral body wall of the arms proximal to the junctions with the central disk region and then were observed. As a positive control experiment ten other starfish were injected with 100 µL of 1 mM S2.

## 3 RESULTS

### 3.1 Mass spectrometric identification of neuropeptides derived from ArPPLNP1 in extracts of *A. rubens* radial nerve cords

ArPPLNP1 is a 224 amino acid protein comprising a predicted 20‐residue signal peptide and five putative SMP‐like peptides (nine copies in total), which we refer to as ArPPLN1a (16 residues; 1 copy), ArPPLN1b (16 residues; 4 copies), ArPPLN1c (17 residues; 2 copies), ArPPLN1d (18 residues; 1 copy) and ArPPLN1e (13 residues; 1 copy) (Figure 1a). The C‐terminal nonapeptide (DPLSAGFTD) of three of these peptides (ArPPLN1a‐c) is identical to the corresponding region of *P. pectinifera* SMP (Kim et al., 2016). All five peptides were detected in extracts of *A. rubens* radial nerve cords using LC‐MS/MS. Four peptides (ArPPLN1a‐d) gave a good series of b fragment ions, as expected for peptides that do not contain a basic C‐terminal amino acid. The fifth peptide (ArPPLN1e) gave nearly complete coverage of b and y ions. The following peptides were observed and selected for MS/MS (Figure 1b–f): ArPPLN1a (GFGMGAYDPLSAGFTD, 811.35 *m/z*, 2+ ion); ArPPLN1b (FGGKGAFDPLSAGFTD, 793.88 *m/z*, 2+ ion); ArPPLN1c (FGGSRGAFDPLSAGFTD, 851.40 *m/z*, 2+ ion), ArPPLN1d (SFVHGDFDPLSTGFVDGD, 956.42 *m/z*, 2+ ion) and ArPPLN1e (AGFMNGVFHPLVA, 680.35 *m/z*, 2+ ion).

### 3.2 The anatomy of the starfish *A. rubens*

**Figure 2:**
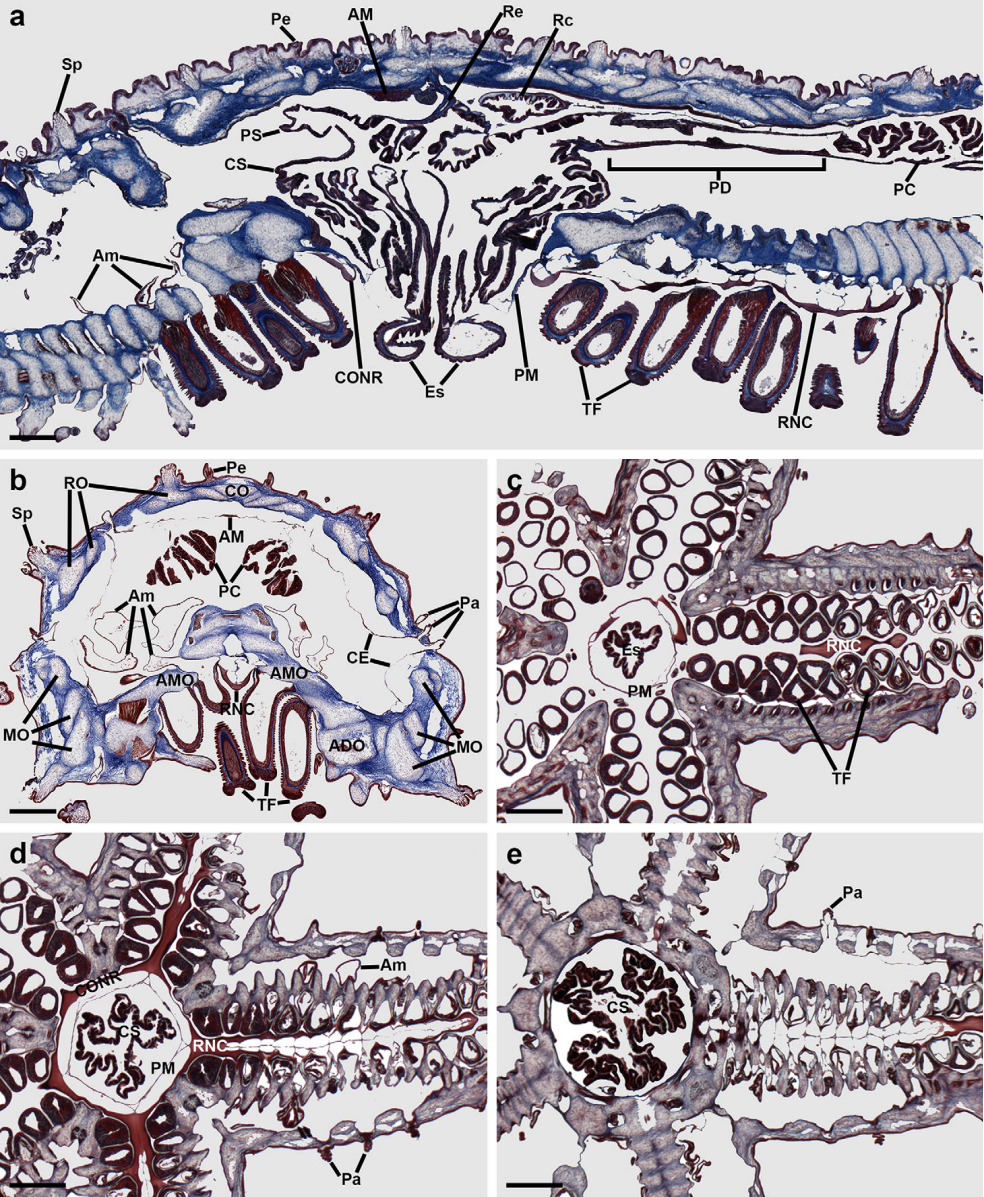
Trichrome stained sections of *A. rubens*. (a) This section runs transversely across the middle of the central disk region and parasagittally through two arms. (b) Transverse section of an arm. (c–e) Horizontal sections through the central disk and arms of a juvenile specimen, with (c) at the level of the esophagus, (d) at the level of the circumoral nerve ring and (e) at the level of the highly folded pouches of the cardiac stomach. Abbreviations: ADO, adambulacral ossicle; AM, apical muscle; Am, ampulla; AMO, ambulacral ossicle; CE, coelomic epithelium; CO, carinal ossicle; CONR, circumoral nerve ring; CS, cardiac stomach; Es, esophagus; MO, marginal ossicles; Pa, papulae; PC, pyloric caeca; PD, pyloric duct; Pe, pedicellariae; PM, peristomial membrane; PS, pyloric stomach; RC, rectal caeca; Re, rectum; RNC, radial nerve cord; RO, reticular ossicles; Sp, spine; TF, tube feet. Scale bars: 500 μm in (a), (b); 200 μm in (c), (d), (e)

To facilitate interpretation of patterns ArPPLNP1 expression in *A. rubens*, as described below, in Figure 2 we show labeled trichrome stained sections of *A. rubens*. Figure 2a shows a section than runs transversely across the middle of the central disk region and parasagittally through two arms. Figure 2b shows a transverse section of an arm. Figure 2c–e show horizontal sections through the central disk and arms of a juvenile specimen.

The nervous system of *A. rubens* comprises radial nerve cords, which are located on the oral side of each arm with two rows of tube feet on either side (Figure 2b, c), and a circumoral nerve ring located in the central disk that links the five radial nerve cords (Figure 2a, d). Linked to each tube foot is an associated contractile ampulla that is located internal to the body wall (Figure 2a, b).

The oral opening to the digestive system (mouth) is surrounded by a contractile peristomial membrane (Figure 2a, c). A short tubular esophagus (Figure 2a, c) links the mouth to a large and highly folded cardiac stomach (Figure 2a, d, e). Aboral to the cardiac stomach is the much smaller pyloric stomach, which is linked to the anus by a short rectum with associated rectal caeca (Figure 2a). Two pyloric caeca are located in each arm (Figure 2a), which are connected to the pyloric stomach by pyloric ducts (Figure 2a).

The body wall comprises calcite ossicles connected by interossicular muscles and collagenous tissue (Figure 2a, b). Externally, the body wall has several appendages that include pedicellariae (Figure 2a, b), spines (Figure 2a, b) and papulae (Figure 2a, b, d, e). Internally, the body wall is lined by a coelomic epithelium (Figure 2a, b) with underlying longitudinally and circularly orientated muscle layers. The longitudinal muscle layer is thickened along the midline of each arm to form the apical muscle (Figure 2a, b).

### 3.3 Localization of ArPPLNP1 transcripts in *A. rubens* using mRNA in situ hybridization

**Figure 3:**
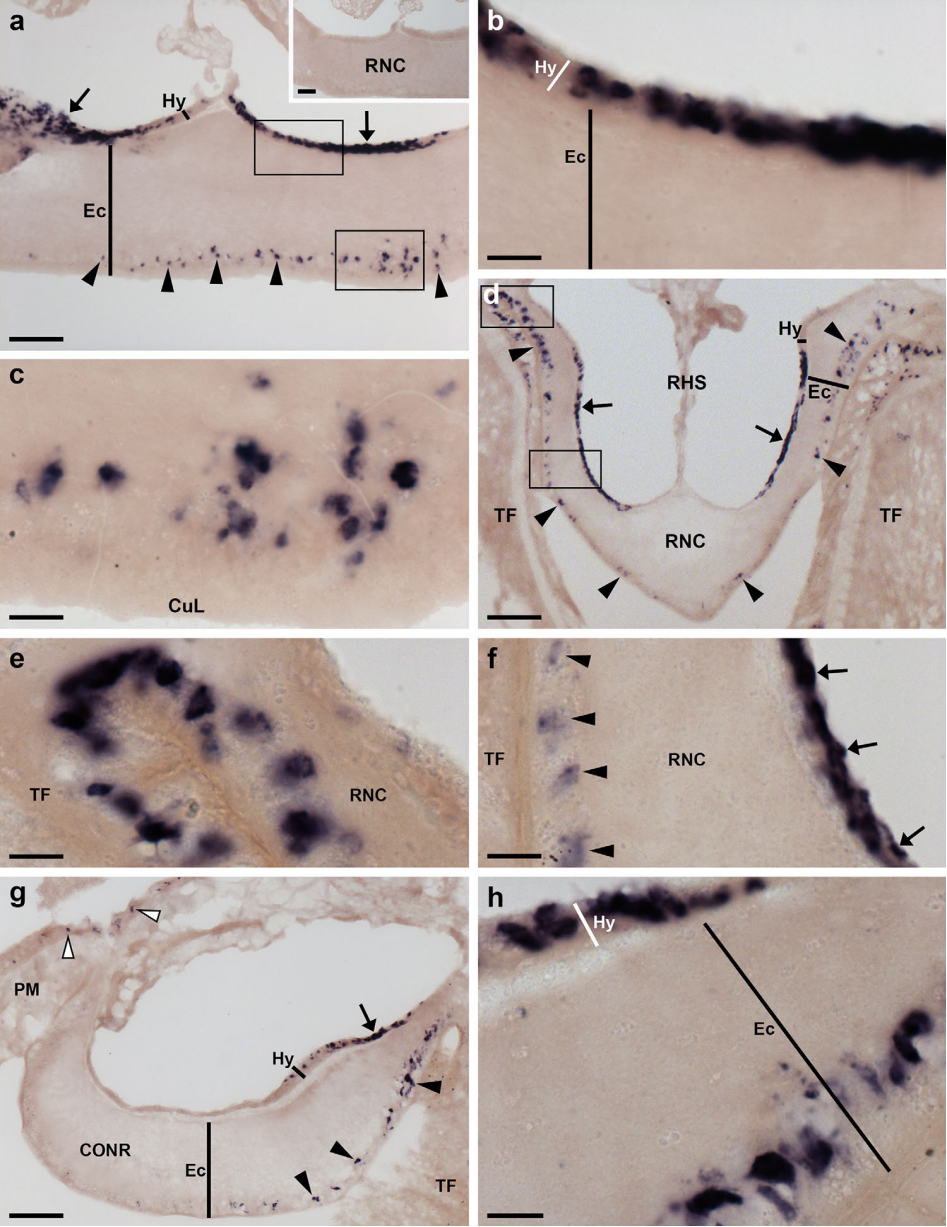
Localization of ArPPLNP1 mRNA in the radial nerve cord and circumoral nerve ring of *A. rubens* using in situ hybridization. (a) Longitudinal parasagittal section of a radial nerve cord incubated with antisense probes showing stained cells in the ectoneural (arrowheads) and hyponeural (arrows) regions. Higher magnification images of the boxed regions are shown in (b) and (c). The inset of panel (a) shows absence of staining in a longitudinal parasagittal section of radial nerve cord incubated with sense probes, demonstrating the specificity of staining observed with antisense probes. (b) High magnification image showing stained cells in the hyponeural region of the radial nerve cord. (c) High magnification image showing stained cells in the ectoneural region of the radial nerve cord. (d) Transverse section of a radial nerve cord showing stained cells in the ectoneural (arrowheads) and hyponeural (arrows) regions. Higher magnification images of the boxed regions are shown in (e) and (f). (e) Stained cells at the junction between the ectoneural region of the radial nerve cord and an adjacent tube foot. (f) Stained cells in the ectoneural (arrowheads) and hyponeural (arrows) regions of the nerve cord. (g) Transverse section of the circumoral nerve ring showing stained cells in the ectoneural (arrowheads) and hyponeural (arrow) regions. Stained cells can also be seen in the coelomic epithelial lining of the peristomial membrane (white arrowheads). (h) High magnification image of the circumoral nerve ring showing stained cells in the ectoneural and hyponeural regions. Abbreviations: CONR, circumoral nerve ring; CuL, cuticular layer; Ec, ectoneural region; Hy, hyponeural region; PM, peristomial membrane; RHS, radial hemal strand; RNC, radial nerve cord; TF, tube foot. Scale bars: 50 μm in (a), (a) inset, (d), (g); 10 μm in (b), (c), (e), (f), (h)

**Figure 4:**
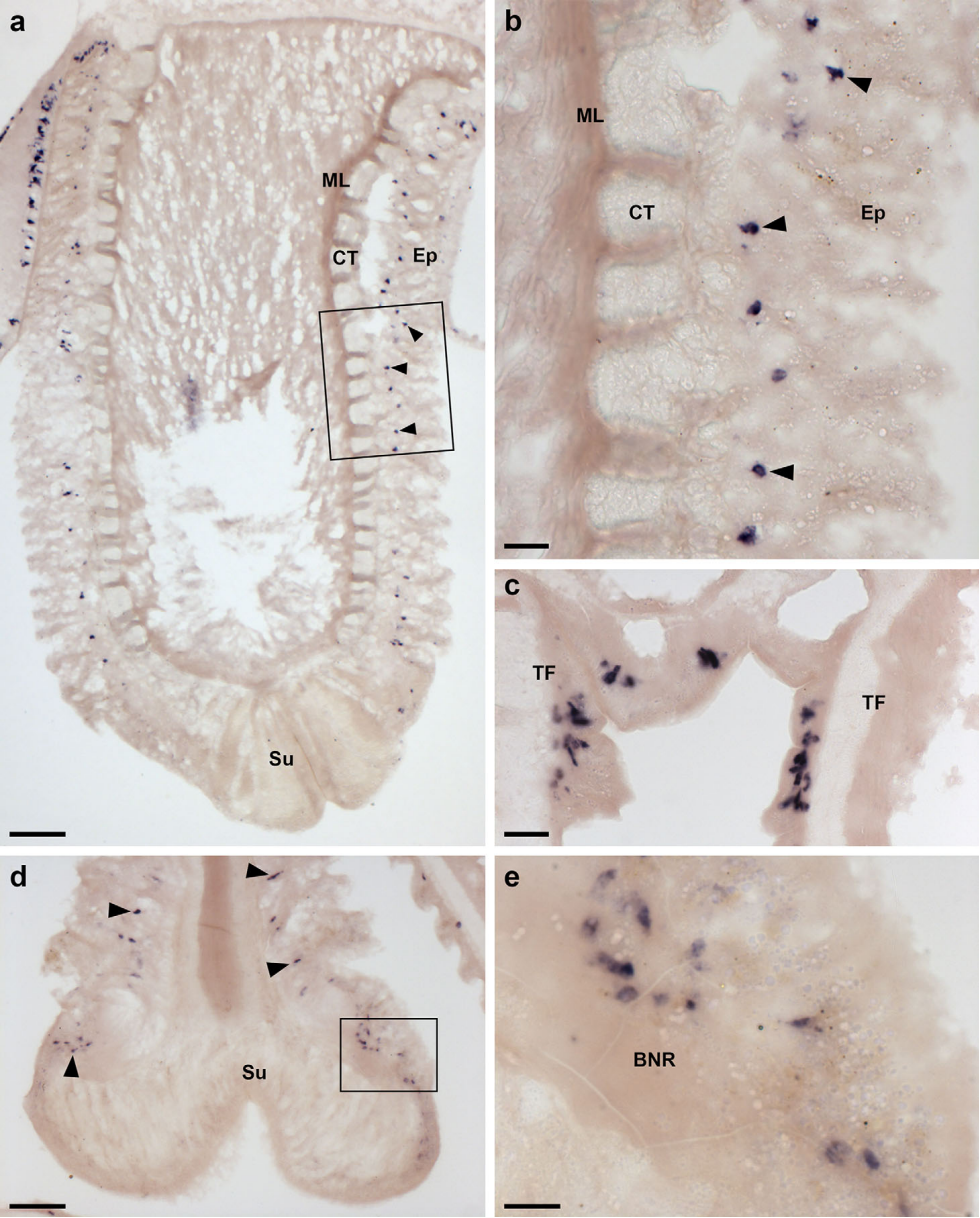
Localization of ArPPLNP1 mRNA in tube feet of *A. rubens* using in situ hybridization. (a, b) Longitudinal section of a tube foot showing stained cells (arrowheads) in the sub‐epithelial layer of the tube foot stem. The boxed region in panel (a) is shown at higher magnification in (b). (c) Stained cells located in the sub‐epithelial layer at the junction between adjacent tube feet. (d) Stained cells (arrowheads) in the sub‐epithelial layer just above the tube foot sucker. The boxed region is shown at higher magnification in (e). (e) Stained cells located near to the tube foot basal nerve ring. Abbreviations: BNR, basal nerve ring; CT, collagenous tissue; Ep, epithelium; ML, muscle layer; Su, sucker; TF: tube foot. Scale bars: 100 μm in (a); 20 μm in (b), (c); 50 μm in (d); 10 μm in (e)

**Figure 5:**
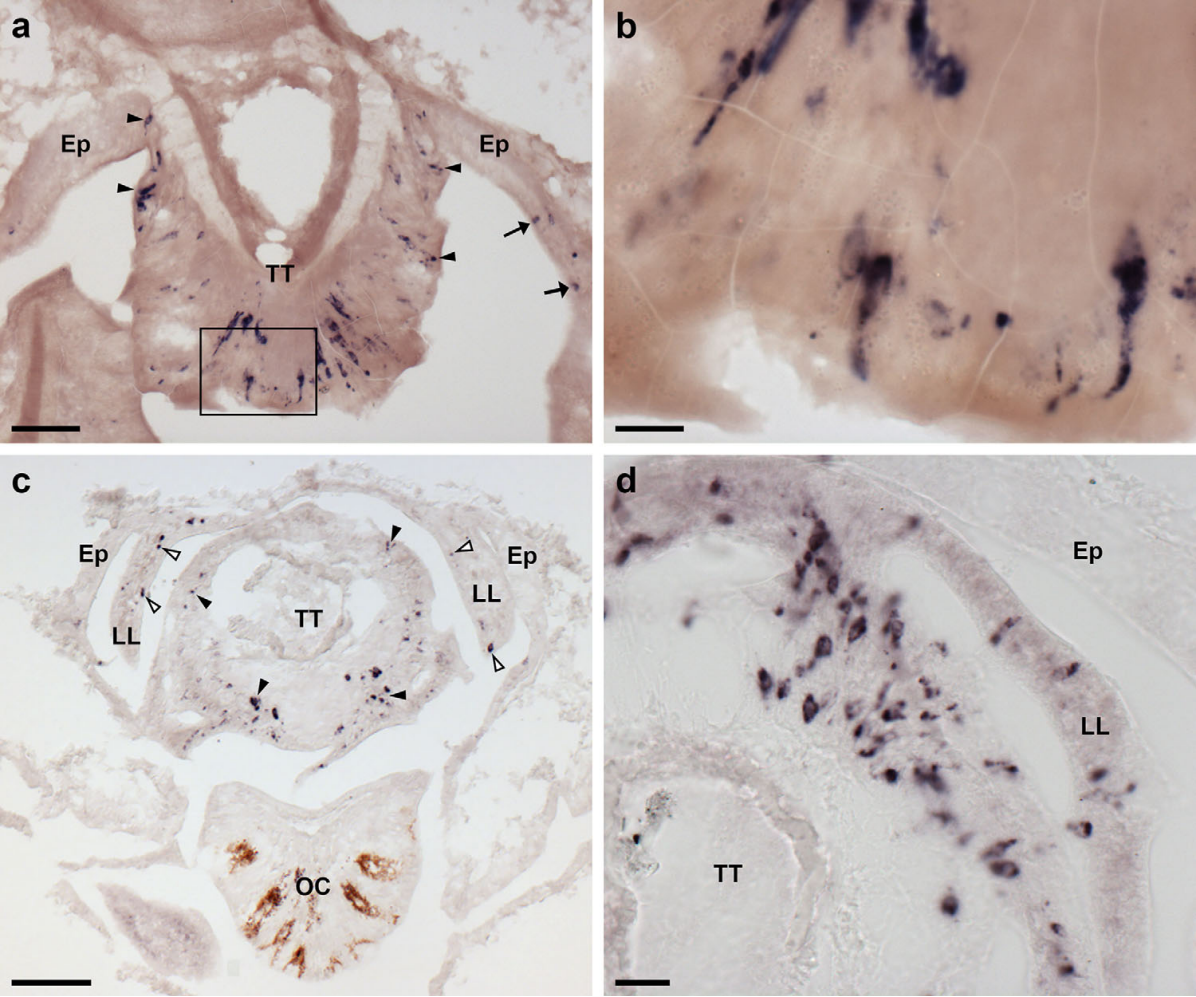
Localization of ArPPLNP1 mRNA in the arm tip region of *A. rubens* using in situ hybridization. (a, b) Transverse section of a paraffin wax embedded arm tip showing the terminal tentacle cut obliquely. Stained cells can be seen in the external epithelial layer of the terminal tentacle (arrowheads) and in the body wall epithelium (arrows) that surrounds the terminal tentacle. The boxed region in (a) is shown at higher magnification in panel (b). (c) Transverse cryostat section of an arm tip showing the pigmented optic cushion and terminal tentacle. Stained cells can be seen in the terminal tentacle external epithelium (black arrowheads) and in the lateral lappets (white arrowheads). (d) A high magnification image of an arm tip showing stained cells in the terminal tentacle and lateral lappet. Abbreviations: Ep, epithelium; LL, lateral lappet; OC, optic cushion; TT, terminal tentacle. Scale bars: 50 μm in (a), (c); 10 μm in (b); 20 μm in (d)

**Figure 6:**
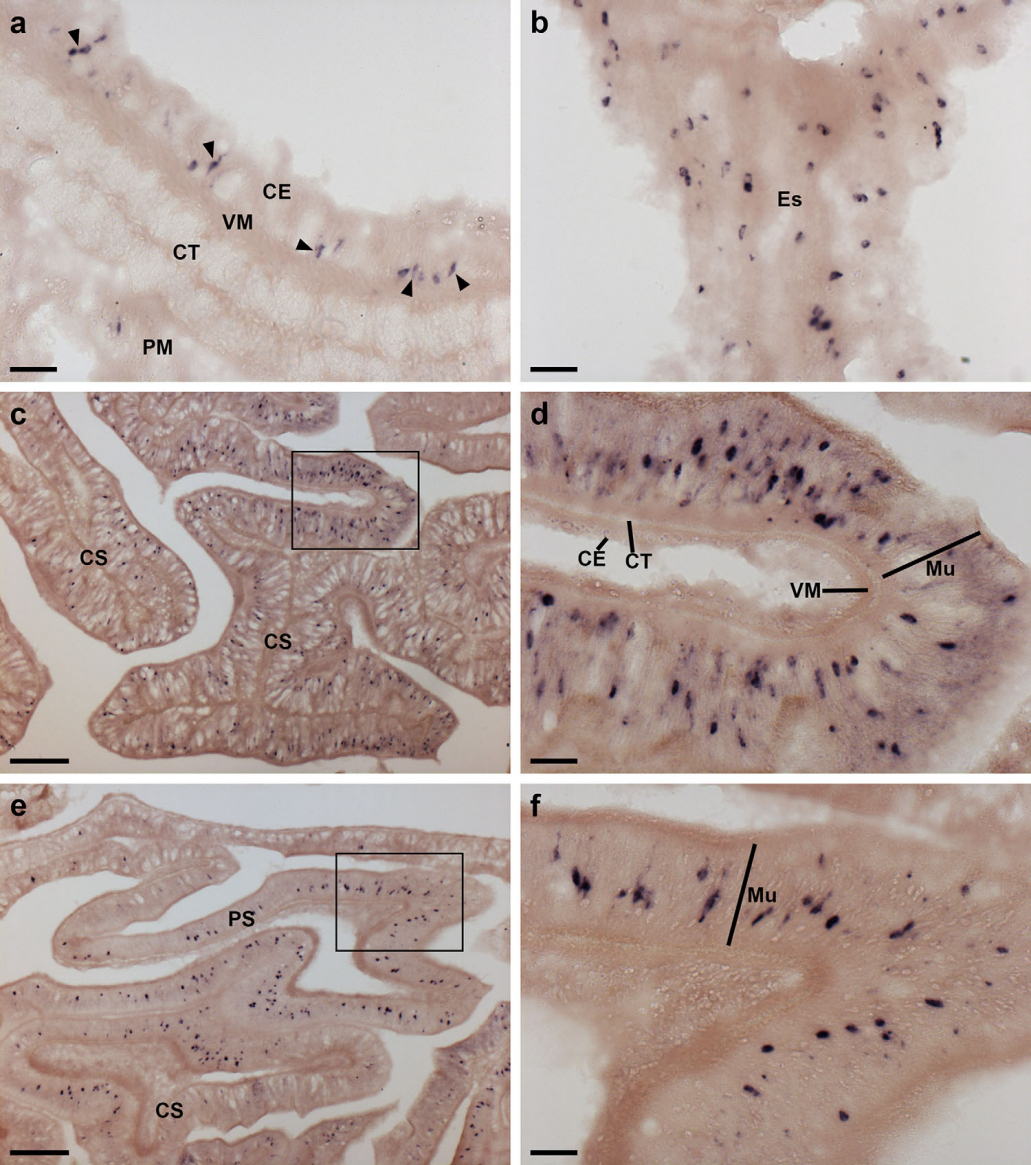
Localization of ArPPLNP1 mRNA in the digestive system of *A. rubens* using in situ hybridization. (a) Transverse section of the peristomial membrane showing stained cells (arrowheads) in the coelomic epithelium. (b) Longitudinal section of the esophagus showing stained cells in the coelomic epithelium. (c, d) Transverse section of the central disk showing stained cells in the cardiac stomach. The boxed region in (c) is shown at higher magnification in panel (d), where stained cells can be seen in mucosal layer. (e, f) Transverse section of the central disk showing stained cells in the cardiac stomach and pyloric stomach. The boxed region of the pyloric stomach in (e) is shown at higher magnification in panel (f), where stained cells can be seen in the mucosal layer. Abbreviations: CE, coelomic epithelium; CS, cardiac stomach; CT, collagenous tissue; Es, esophagus; Mu, mucosa; PM, peristomial membrane; PS, pyloric stomach; VM, visceral muscle. Scale bars: 20 μm in (a), (b), (d), (f); 100 μm in (c), (e)

**Figure 7:**
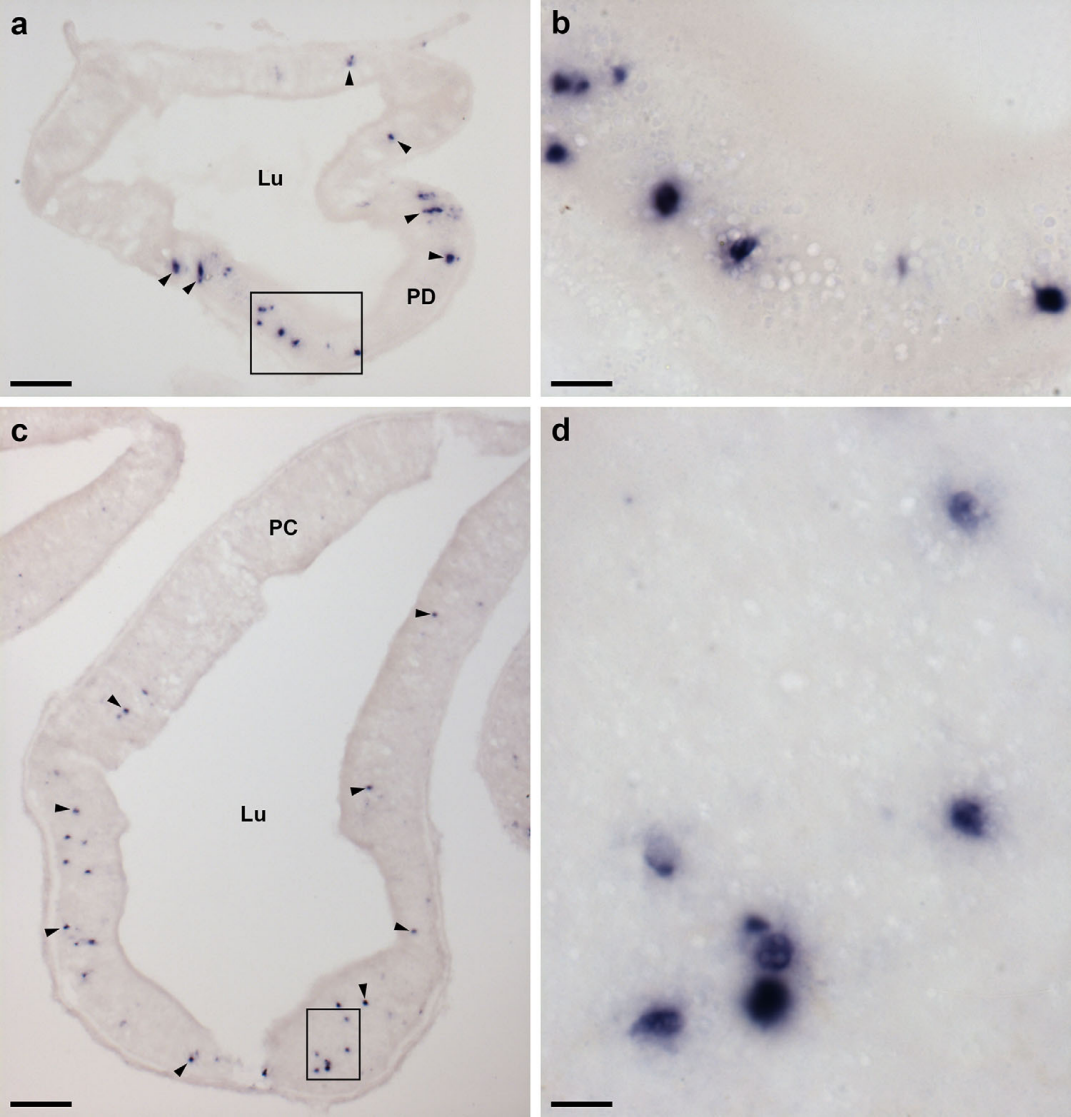
Localization of ArPPLNP1 mRNA in the pyloric ducts and pyloric caeca of *A. rubens* using in situ hybridization. (a, b) Transverse section of a pyloric duct showing stained cells (arrowheads) that are concentrated in the oral (lower) domain of the duct. The boxed region in (a) is shown at higher magnification in panel (b), where stained cells can be seen in the mucosal layer. (c, d) Transverse section of a pyloric caecum showing stained cells (arrowheads) that are concentrated in the oral (lower) domain of a pyloric caecum diverticulum. The boxed region in (c) is shown at higher magnification in panel (d), where stained cells can be seen in the mucosal layer. Abbreviations: Lu, lumen; PC, pyloric caeca; PD, pyloric duct. Scale bars: 50 μm in (a); 10 μm in (b), (d); 100 μm in (c)

**Figure 8:**
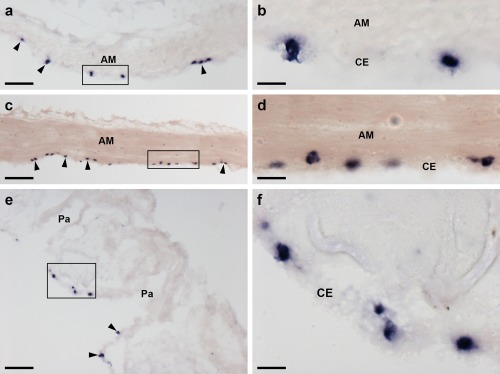
Localization of ArPPLNP1 mRNA in the apical muscle and coelomic lining of the body wall in *A. rubens* using in situ hybridization. (a, b) Transverse section of apical muscle showing stained cells (arrowheads) in the coelomic epithelium. The boxed region in (a) is shown at higher magnification in panel (b). (c, d) Longitudinal section of apical muscle showing stained cells (arrowheads) in the coelomic epithelium. The boxed region in (c) is shown at higher magnification in panel (d). (e, f) Transverse section of an arm showing stained cells (arrowheads) in the coelomic epithelium at the base of a papula. The boxed region in (e) is shown at higher magnification in panel (f). Abbreviations: AM, apical muscle; CE, coelomic epithelium; Pa, papulae. Scale bars: 50 μm in (a), (c), (e); 10 μm in (b), (d), (f)

Analysis of the distribution of ArPPLNP1 transcripts in *A. rubens* using mRNA in situ hybridization revealed a widespread pattern of expression, including stained cells in the radial nerve cords and circumoral nerve ring that form the main tracts of the nervous system (Figure 3), the tube feet (Figure 4), the terminal tentacle (Figure 5), the digestive system (Figure 6, 7) and the ceolomic epithelial lining of the body wall (Figure 8).

In longitudinal sections of the radial nerve cord, stained cells can be seen in the hyponeural region (Figure 3a, b) and along the length of the ectoneural region in the sub‐cuticular epithelium (Figure 3a, c). The specificity of the staining observed with antisense probes (Figure 3a) was demonstrated by control experiments where no staining was observed with sense probes (Figure 3a inset). Transverse sections of the radial nerve cords revealed that the stained cells in the ectoneural region are concentrated in the lateral regions of the radial nerve cords (Figure 3d) and this population of cells extends into the epithelium of adjacent tube feet (Figure 3e). In contrast, stained cells are only sparsely distributed in the ectoneural epithelium at the apex of the V‐shaped nerve cord (Figure 3d). By way of comparison, a dense population of stained cells is present throughout much of the hyponeural region of the radial nerve cord (Figure 3a, b, f). The pattern of expression in the circumoral nerve ring is consistent with that seen in the radial nerve cords. Thus, cells are present in both the hyponeural and ectoneural regions; however, in the ectoneural epithelium stained cells are concentrated in the region adjacent to the perioral tube feet and are sparser in the region adjacent to the peristomial membrane (Figure 3g, h).

In the tube feet, which enable locomotor activity in starfish, stained cells are present in a basiepithelial position at the junction between the tube feet and the radial nerve cord or circumoral nerve ring (Figure 4a), along the length of the tube foot stem (Figure 4a, b) and at the junction between adjacent tube feet (Figure 4c). Stained cells are also present in the tube foot sucker, in close proximity to the basal nerve ring (Figure 4d, e).

The terminal tentacle is a non‐locomotory but tube foot‐like sensory organ located at the tip of each starfish arm and, consistent with the expression of ArPPLNP1 in tube feet, stained cells are also present in the terminal tentacle (Figure 5). Stained cells are present in the wall of the terminal tentacle (Figure 5) and in lateral lappets (Figure 5c, d), flaps of epithelial tissue that hang down from the body wall on both sides of the terminal tentacle. Stained cells are also present in the body wall epithelium that forms the roof and wall of the cavity containing the terminal tentacle (Figure 5a). However, no stained cells were observed in the pigmented photosensory optic cushion, which is located at the base of the terminal tentacle (Figure 5c).

In the digestive system, ArPPLNP1 mRNA expression was detected in the peristomial membrane (Figure 6a), esophagus (Figure 6b), cardiac stomach (Figure 6c, d), pyloric stomach (Figure 6e, f), pyloric ducts (Figure 7a, b) and pyloric caeca (Figure 7c, d). In the peristomial membrane and esophagus, stained cells are present in the coelomic epithelium and in the external epithelium (Figure 6a, b). In the cardiac stomach, ArPPLNP1 is widely expressed. Stained cells are present in the mucosa of the floor (Figure 6c), pouches and roof (Figure 6c, d) of the cardiac stomach. The floor of the pyloric stomach has a similar pattern of expression as the roof of the cardiac stomach (Figure 6e, f).

The pyloric ducts, which link the pyloric stomach with the paired pyloric caeca in each arm, contain stained cells that are concentrated in the mucosal epithelium on the oral side of the ducts (Figure 7a, b). Similarly, stained cells are concentrated in the mucosal epithelium on the oral side of the central canal of the pyloric caeca (Figure 7c, d), which is directly connected to the pyloric ducts (Anderson, 1953).

ArPPLNP1‐expressing cells are present within the aboral coelomic epithelium. Stained cells can be seen associated with the longitudinally orientated apical muscle, which is shown here in both transverse (Figure 8a, b) and longitudinal (Figure 8c, d) sections of the starfish arm. Stained cells can also be seen in the coelomic epithelium located at the base of the finger‐like papulae, which project through the body wall to the external aboral surface of the arms (Figure 8e, f).

### 3.4 Characterisation of a rabbit antiserum to ArPPLN1b using ELISA

**Figure 9:**
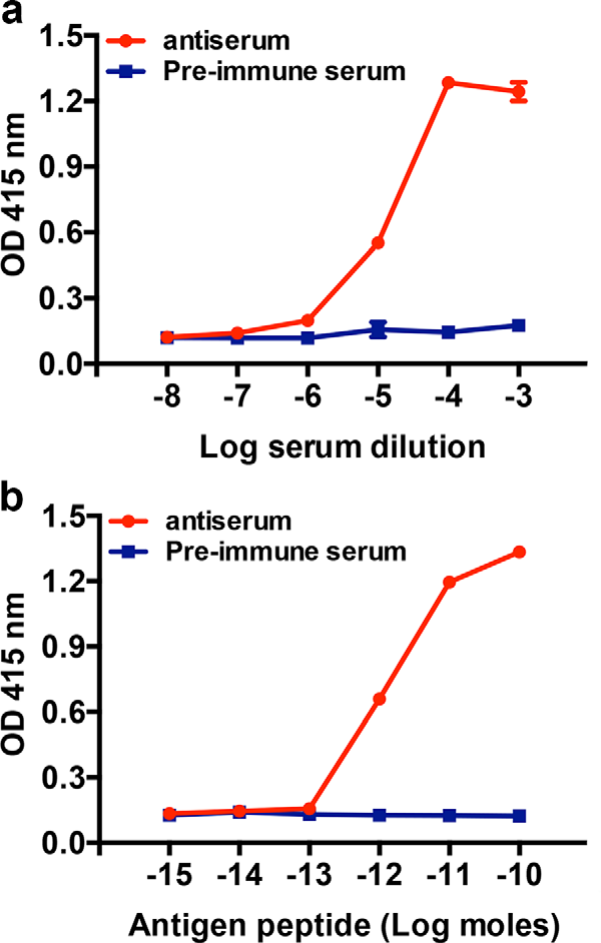
Characterization of rabbit antiserum to ArPPLN1b using an enzyme‐linked immunosorbent assay (ELISA). (a) Incubation of antiserum (red) and pre‐immune serum (blue) at dilutions between 10^−3^ and 10^−8^ with 0.1 nmol of antigen peptide (ArPPLN1‐ag) per well reveals no immunoreaction with pre‐immune serum, whereas with the antiserum the antigen is detected at well above the background optical density (OD) with dilutions from 10^−3^ to 10^−5^. (b) Incubation of antiserum (red) and pre‐immune serum (blue) at 10^−4^ dilution with between 10^−15^ and 10^−10^ moles of antigen peptide (ArPPLN1‐ag) per well reveals no immunoreaction with pre‐immune serum, whereas with the antiserum the antigen is detected at well above the background OD with 10^−12^ to 10^−10^ moles per well. All data points are mean values from two separate experiments performed in duplicate

To assess the presence and titre of antibodies to the ArPPLN1‐ag peptide in rabbit sera, antiserum and pre‐immune serum were incubated at a range of dilutions (10^−3^–10^−8^) with a fixed amount of the antigen peptide added to each well of the microtitre plate (10^−10^ moles). As expected, no ArPPLN1‐ag immunoreactivity was detected with pre‐immune serum but ArPPLN1‐ag immunoreactivity was detected with antiserum at dilutions ranging from 10^−3^ to 10^−5^ (Figure 9a). To assess the sensitivity of the antiserum for detection of the ArPPLN1‐ag peptide, the antiserum (diluted to 10^−4^) was incubated with different amounts of peptide ranging from 10^−10^ to 10^−15^ moles. At this antiserum dilution, the peptide was detected in the range from 10^−10^ to 10^−12^ moles (Figure 9b). Collectively, these data indicate that the antiserum contains a high titre of antibodies to the ArPPLN1‐ag peptide.

### 3.5 Immunohistochemical localization of neuropeptides derived from ArPPLNP1 in *A. rubens*

#### 3.5.1 Radial nerve cords, circumoral nerve ring, marginal nerve cords and lateral motor nerves

Antiserum to the C‐terminal region of ArPPLN1b (KGAFDPLSAGFTD; see above) was used to investigate the distribution of this peptide in *A. rubens* using immunohistochemistry. It should be noted, however, that two of the other peptides derived from ArPPLNP1 (ArPPLN1a and ArPPLN1c) have the same C‐terminal DPLSAGFTD sequence as ArPPLN1b and so it is likely that these are also immunoreactive with the antiserum. Having demonstrated using ELISA that the antiserum contains a high titre of antibodies to the antigen peptide (Figure 9), the antiserum was used for immunohistochemistry at a dilution of 10^−5^.

**Figure 10:**
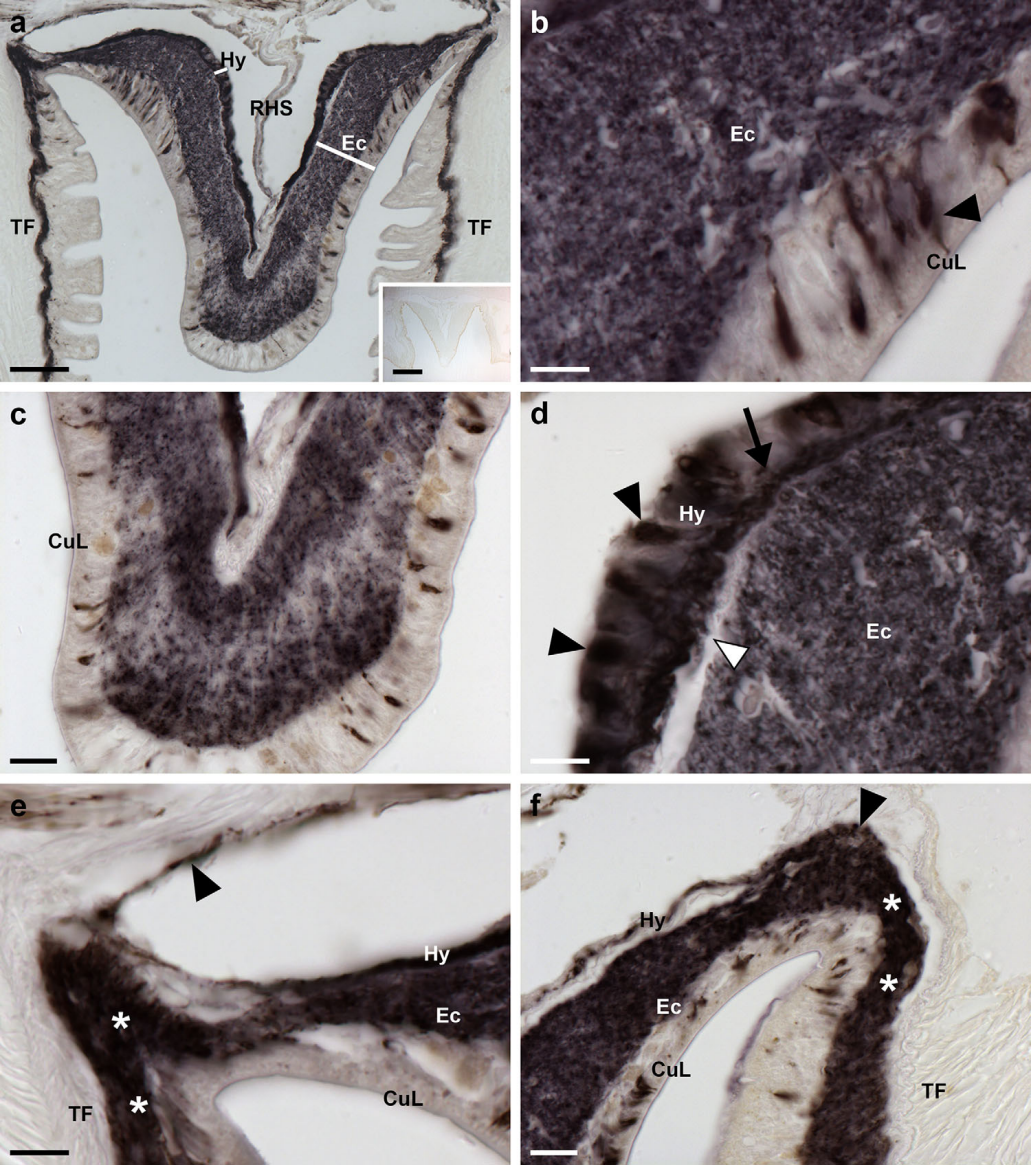
Localization of ArPPLN1b‐immunoreactivity (ArPPLN1b‐ir) in the radial nerve cord of *A. rubens*. (a) Tranvserse section of a radial nerve cord showing ArPPLN1b‐ir in both the ectoneural and hyponeural regions. The inset of (a) shows absence of immunostaining in a radial nerve cord section incubated with ArPPLN1b antiserum pre‐absorbed with the antigen peptide (ArPPLN1b‐ag), demonstrating the specificity of immunostaining observed with the ArPPLN1b antiserum. (b) High magnification image of the ectoneural region of the radial nerve cord showing immunostained bipolar shaped cells in the sub‐cuticular epithelium (arrowhead) and densely packed immunostained processes in the underlying neuropile region. (c) High magnification image of the ectoneural region at the tip of the V‐shaped radial nerve cord showing layer‐specific variation in the density of immunostained processes. (d) High magnification image showing immunostained monopolar shaped cells (arrowheads) and their stained processes (arrow) in the hyponeural region of the radial nerve cord. The unstained collagenous tissue layer (white arrowhead) that separates the hyponeural region from the densely stained ectoneural neuropile can also be seen here. (e, f) High magnification images showing immunostaining at the junction between the radial nerve cord and an adjacent tube foot. The continuity of immunostaining in the ectoneural region of the radial nerve and in the basiepithelial nerve plexus of the tube foot (asterisks) can be seen here. In (e) the stained process(es) (arrowhead) of a hyponeural neuron(s) can be seen projecting over the roof of the perihemal canal in close association with the transverse infra‐ambulacral muscle. In (f) the stained processes (arrowhead) of hyponeural neurons can be seen projecting to the base of the adjacent tube foot. Abbreviations: CuL, cuticle layer; Ec, ectoneural region; Hy, hyponeural region; RHS, radial hemal strand; TF, tube foot. Scale bars: 50 μm in (a); 200 μm in (a) inset; 10 μm in (b), (d), (e); 20 μm in (c), (f)

An extensive and intense pattern of ArPPLN1b‐immunoreactivity (ir) was revealed in radial nerve cord of *A. rubens* (Figure 10a). The specificity of this immunostaining was confirmed in control experiments testing antiserum that had been pre‐absorbed with the antigen peptide. Thus, in tests with pre‐absorbed antiserum no immunostaining was observed in the radial nerve cords (Figure 10a inset) or in other parts of the starfish body (data not shown).

ArPPLN1b‐ir was revealed in bipolar cell bodies located in the epithelium of the ectoneural region of the radial nerve cord and in a dense meshwork of fibers in the underlying neuropile (Figure 10a, b). Interestingly, the intensity of immunostaining in the ectoneural neuropile is not homogeneous; for example, in the region that forms the tip of the V‐shaped nerve cord there is zone of neuropile with a lower density of stained fibers that is bounded above (aboral) and below (oral) by zones with a higher density of stained fibers (Figure 10a, c). ArPPLN1b‐ir was also revealed in monopolar cell bodies in the hyponeural region of the radial nerve cord and the axonal processes of these neurons (Figure 10a, d), which can be seen projecting to the base of the adjacent tube foot stem and to the transverse infra‐ambulacral muscle (Figure 10e). Immunostained processes in the neuropile of the ectoneural region of the radial nerve cord are continuous with the basiepithelial nerve plexus of the adjacent tube foot Figure 10f).

**Figure 11:**
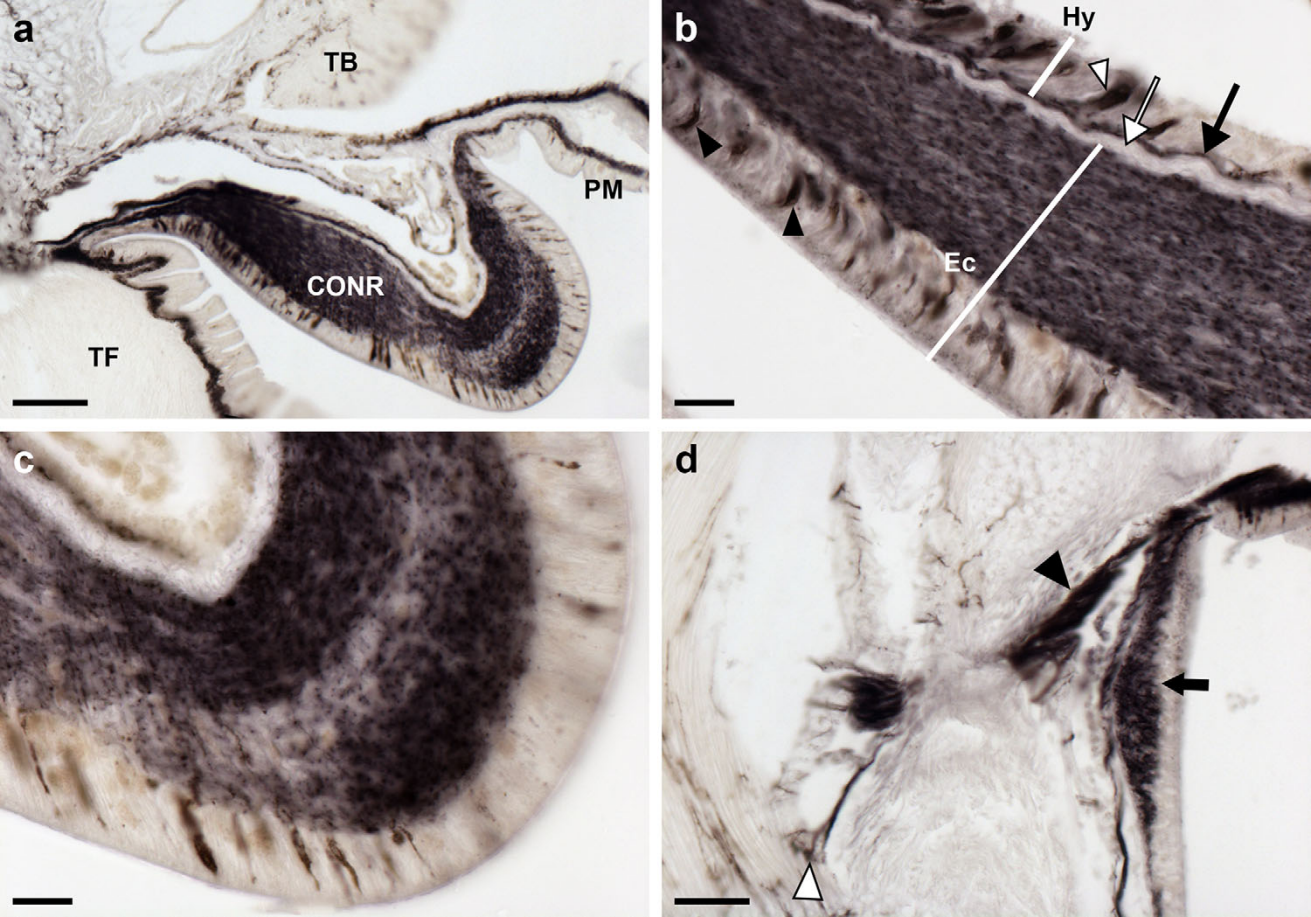
Localization of ArPPLN1b‐immunoreactivity in the circumoral nerve ring and marginal nerve of *A. rubens*. (a) ArPPLN1b‐ir in a transverse section of the circumoral nerve ring; here immunostained processes can also be seen in the peristomial membrane, in an adjacent oral tube foot and in a Tiedemann's body. (b) A high magnification image of the circumoral nerve ring showing immunostained bipolar shaped cells in the sub‐cuticular epithelium (arrowheads) of the ectoneural region and densely packed immunostained processes in the underlying neuropile. In the hyponeural region immunostained monopolar shaped cells (white arrowhead) can be seen with their stained processes (arrow) adjacent to the unstained collagenous tissue layer (white arrow). (c) High magnification image of the ectoneural region of the circumoral nerve ring showing layer‐specific variation in the density of immunostained processes. (d) Immunostaining in a sub‐epithelial thickening of the basiepithelial plexus known as the marginal nerve (arrow), which is located at the junction between the outer row of tube feet and the adjacent body wall. Internal to the marginal nerve, separated by a thin layer of collagenous tissue, can be seen stained axonal processes that coalesce to form the lateral motor nerve (arrowhead). Individual stained axonal processes derived from the lateral motor nerve can be seen here innervating an adjacent interossicular muscle (white arrowhead). Abbreviations: CONR, circumoral nerve ring; Ec, ectoneural region; Hy, hyponeural region; PM, peristomial membrane; TB, Tiedemann's body; TF, tube foot. Scale bars: 100 μm in (a); 20 μm in (b), (c); 50 μm in (d)

The pattern of immunostaining in the circumoral nerve ring (Figure 11a, b) is consistent with that in the radial nerve cords, with bipolar cell bodies in the ectoneural epithelium projecting into the densely stained underlying neuropile and with the processes of stained monopolar cells in the hyponeural region projecting into underlying nerve tract that is separated from the ectoneural neuropile by a thin layer of unstained collagenous tissue (Figure 11b, c). As in the radial nerve cord, there was regional variability in the density of immunostained fibers in the ectoneural neuropile. Thus, in the region of the circumoral nerve ring proximal to the peristomial membrane there is a layer of neuropile with a lower density of immunostained fibers that is bounded on either side by layers of neuropile with a higher density of immunostained fibers (Figure 11c). This pattern of staining seen in the circumoral nerve ring is anatomically equivalent to the “banded” pattern of staining seen in the tip region of the radial nerve cord (see above). Stained fibers in the ectoneural neuropile are continuous with the basiepithelial plexus of the adjacent peristomial membrane (Figure 11a).

Located lateral to the outer row of tube feet on each side of the arm is the marginal nerve, which is a thickening of the basiepithelial nerve plexus (Smith, 1937, 1950). Cells exhibiting ArPPLN1b‐ir are present in the epithelial layer of the marginal nerve and ArPPLN1b‐ir is present in the underlying neuropile (Figure 11d). Closely associated with the marginal nerves are the lateral motor nerves, which contain the axons of hyponeural motor neurons that innervate the body wall musculature and the coelomic lining of the aboral body wall (Smith, 1950). ArPPLN1b‐ir is present in the lateral motor nerves and stained processes emerging from a lateral motor nerve can be seen innervating the muscle that links the ambulacral and adambulacral ossicles (Figure 11d).

#### 3.5.2 Hemal system and Tiedemann's bodies

**Figure 12:**
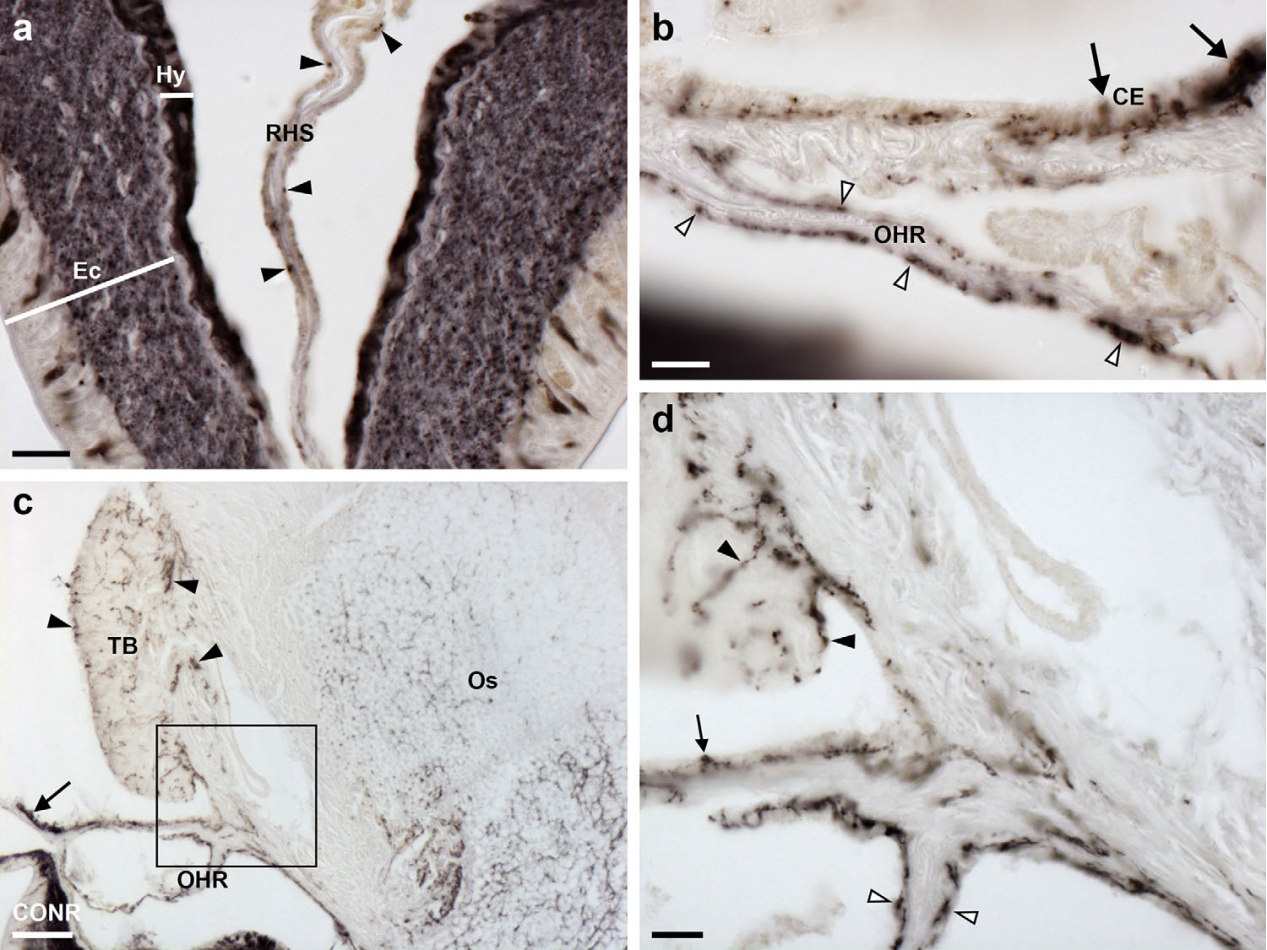
Localization ArPPLN1b immunoreactivity in the hemal system and Tiedemann's bodies of *A. rubens*. (a) Immunostained processes (arrowheads) are present in the radial hemal strand, which is connected to the intensely stained radial nerve cord. (b) Immunostained processes (white arrowheads) are present in the oral hemal ring, which is connected to the circumoral nerve ring (out of focus at bottom left). Immunostained processes can also be seen here in the coelomic epithelium (arrows). (c, d) Immunostained processes in one of the ten Tiedemann's bodies, which are located aboral to the circumoral nerve ring in the central disk. Immunostained processes can also be seen on the right side of the image, where they are associated with interossicular muscles of the body wall (see Figure 18). The boxed region in (c) is shown at higher magnification in panel (d), where immunostained processes can be seen in the Tiedemann's body (black arrowheads), the coelomic epithelium (arrow) and the oral hemal ring (white arrowheads). Abbreviations: CE, coelomic epithelium; CONR, circumoral nerve ring; Ec, ectoneural region; Hy, hyponeural region; OHR, oral hemal ring; Os, ossicle; RHS, radial hemal strand; TB, Tiedemann's body. Scale bars: 20 μm in (a), (b), (d); 100 μm in (c)

Anatomically associated with the radial nerve cords and circumoral nerve ring are the radial hemal strands and the oral hemal ring, respectively, which constitute, at least in part, the circulatory system of starfish (Ferguson, 1984). ArPPLN1b‐ir is present in fibers located in the walls of the radial hemal strands and the oral hemal ring (Figure 12a, b). Forming a completely separate system of fluid‐filled canals in starfish are the radial water canals and ring canal, components of the water vascular system that are in continuity with the fluid‐filled lumen of the tube feet and ampullae (Smith, 1950). Closely associated with the ring canal are five pairs of organs known as Tiedemann's bodies, which also contain fibers that exhibit ArPPLN1b‐ir (Figure 12c, d).

#### 3.5.3 Tube feet and terminal tentacle

**Figure 13:**
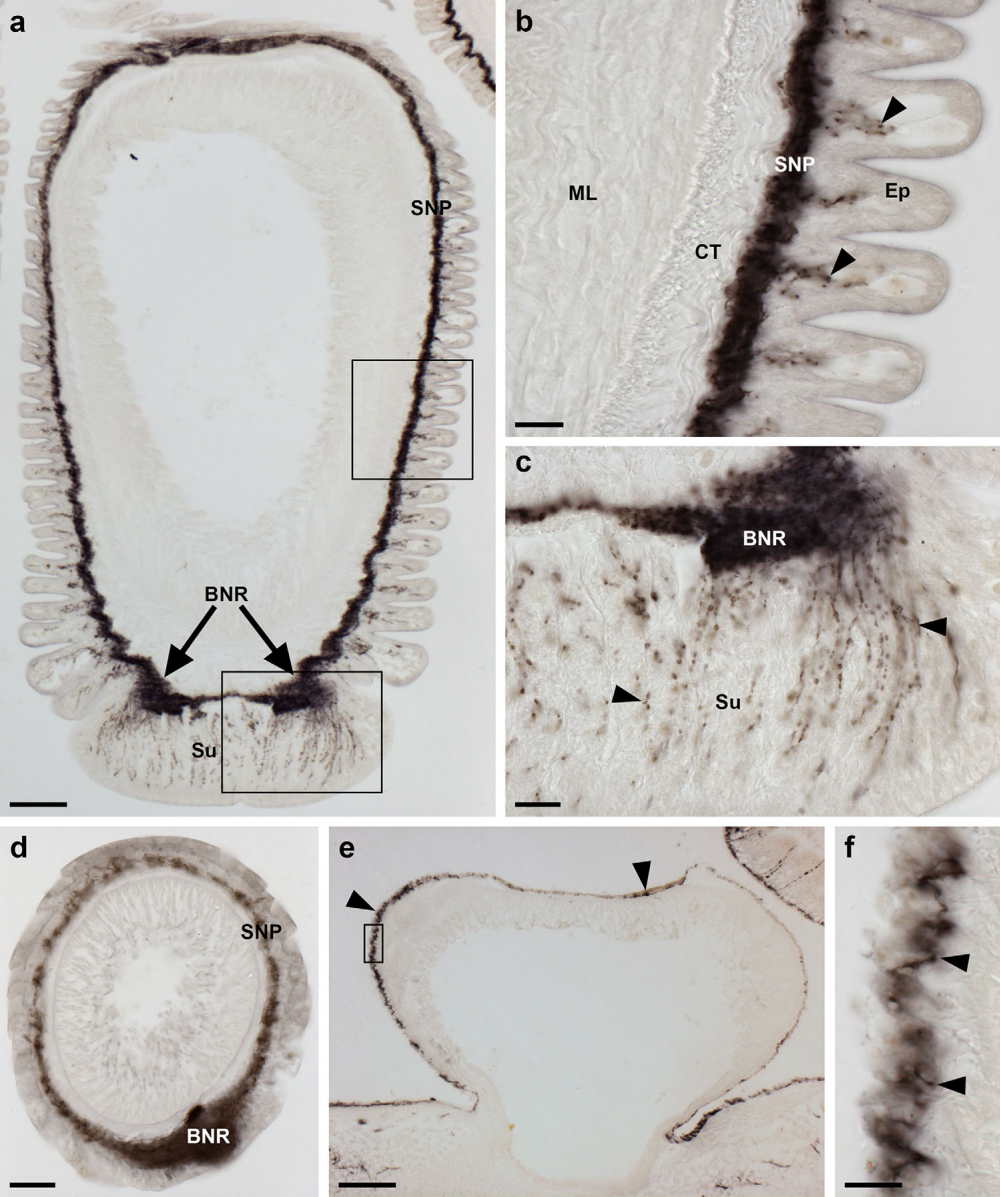
Localization of ArPPLN1b immunoreactivity in tube feet and ampullae of *A. rubens*. (a) Longitudinal section of a tube foot showing immunostaining in the sub‐epithelial nerve plexus, basal nerve ring and sucker. The boxed regions are shown at higher magnification in panels (b) and (c). (b) High magnification image showing immunostaining in the sub‐epithelial nerve plexus and in processes projecting into epithelial folds of a contracted tube foot (arrowheads). (c) High magnification image showing immunostaining in the basal nerve ring and in processes projecting into the tube foot sucker (arrowheads). (d) Immunostaining in a transverse section of a tube foot showing immunostaining in the sub‐epithelial nerve plexus and basal nerve ring. (e, f) Immunostaining in the sub‐epithelial nerve plexus of an ampulla (arrowheads). The boxed region in panel (e) is shown at higher magnification in panel (f). Abbreviations: BNR, basal nerve ring; CT, collagenous tissue; Ep, epithelium; ML, muscle layer; SNP, sub‐epithelial nerve plexus; Su, sucker. Scale bars: 100 μm in (a), (e); 20 μm in (b), (c), (d); 10 μm in (f)

Strong immunostaining is present throughout the basiepithelial nerve plexus of the tube foot stem (Figure 13a, b) and in the basal nerve ring located in the tube foot sucker (Figure 13c, d). Immunostained processes can be seen extending from the basiepithelial nerve plexus into each fold of the contracted tube foot stem (Figure 13b). Likewise, immunostained processes extend from the basal nerve ring into the sucker (Figure 13c). Immunostained processes are also present beneath the coelomic epithelium of the tube foot ampulla (Figure 13e, f).

**Figure 14:**
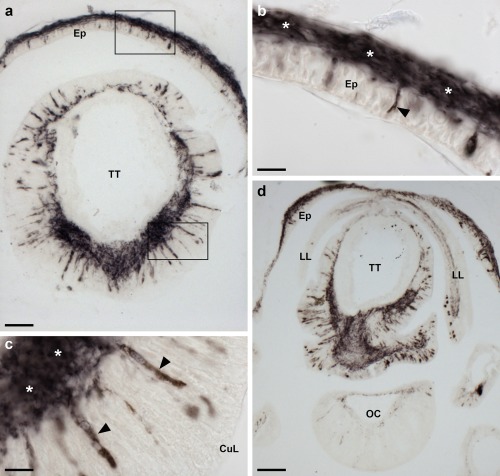
Localization of ArPPLN1b immunoreactivity in the terminal tentacle and associated structures in the arm tip of *A. rubens*. (a) Immunostaining in a transverse section of a terminal tentacle and in the surrounding body wall epithelium. The boxed regions are shown at higher magnification in panel (b) and (c). (b) A high magnification image of arm tip body wall epithelium showing immunostained bipolar shaped cells (arrowhead) and in a dense meshwork of fibers in the underlying basiepithelial nerve plexus (asterisks). (c) High magnification image of the terminal tentacle showing immunostained bipolar shaped cells in the epithelium (arrowheads) and in a dense meshwork of fibers in the underlying basiepithelial nerve plexus (asterisks). (d) Immunostaining in a transverse section at the base of the terminal tentacle. Immunostained cells and processes can be seen here in the body wall epithelium, the terminal tentacle and associated lateral lappets and the optic cushion. Abbreviations: CuL, cuticle layer; Ep, epithelium; LL, lateral lappet; OC, optic cushion; TT, terminal tentacle. Scale bars: 50 μm in (a); 10 μm in (b), (c); 100 μm in (d)

In the terminal tentacle, immunostained bipolar‐shaped cells are present in the epithelial wall of this organ and in the underlying basiepithelial nerve plexus. Stained cells and underlying processes are also present in the optic cushion, the lateral lappets of the terminal tentacle and in the body wall epithelium that surrounds the cavity containing the terminal tentacle (Figure 14a–d).

#### 3.5.4 Digestive system

**Figure 15:**
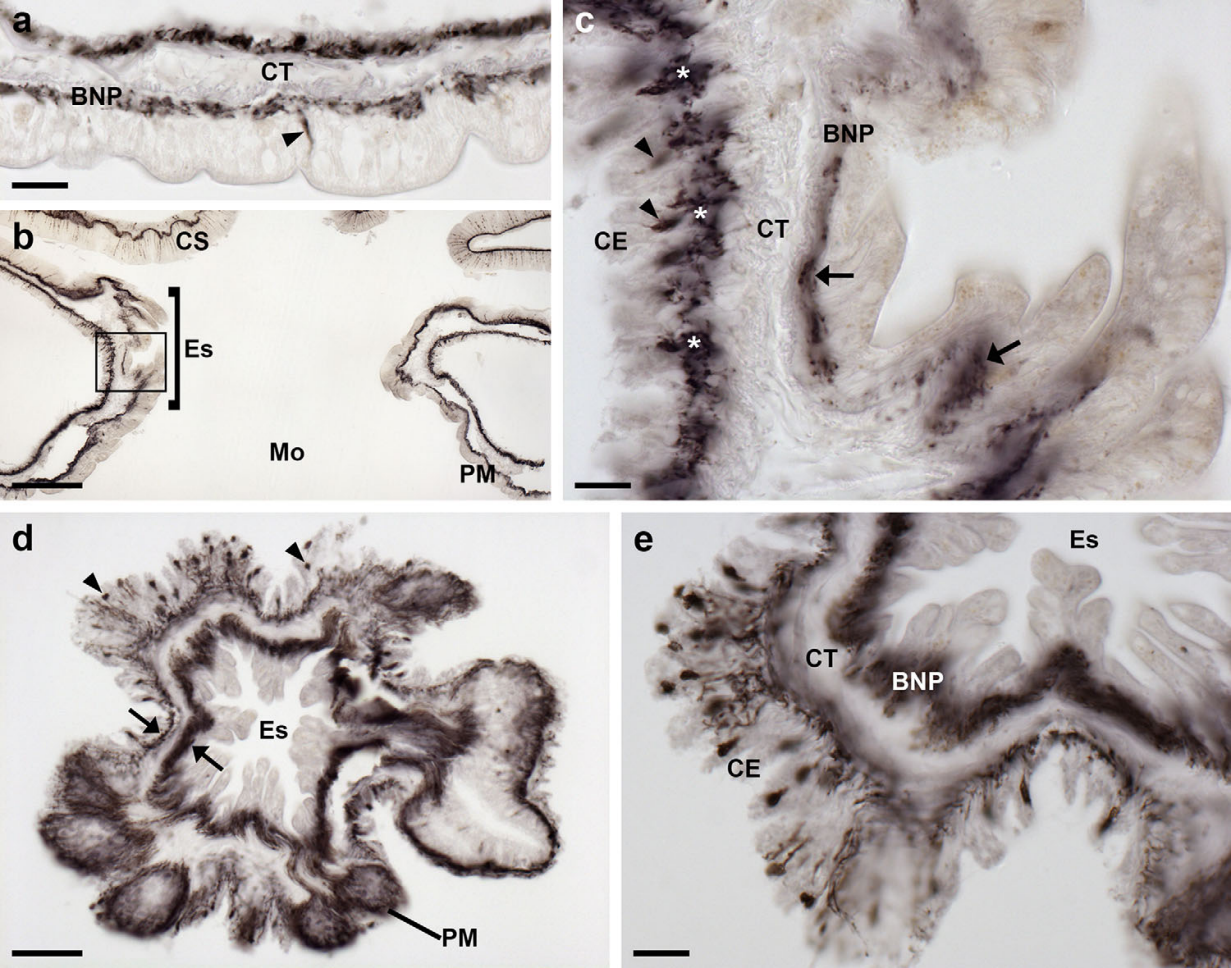
Localization of ArPPLN1b immunoreactivity in the peristomial membrane and esophagus of *A. rubens*. (a) An immunostained bipolar shaped cell can be seen here in the external epithelial layer of the peristomial membrane (arrowhead) and in processes located in the underlying basiepithelial nerve plexus. Immunostaining is also present in cells and processes located beneath the coelomic epithelium, which is separated from the basiepithelial nerve plexus by a layer of collagenous tissue. (b) Transverse section of the central disk showing immunostaining in the peristomial membrane, the esophagus and the cardiac stomach. The boxed region is shown at higher magnification in panel (c). (c) Longitudinal section of the esophagus (in a transverse section of the central disk) showing immunostained cells (arrowheads) and processes (asterisks) beneath the coelomic epithelium and stained processes (arrows) in the basiepithelial nerve plexus beneath the epithelial layer that forms the external lining of the esophagus. (d) Horizontal section of the central disk at the level of the junction between the esophagus and the peristomial membrane showing immunostained cells (arrowheads) and processes (arrows). (e) High magnification transverse section of the esophagus (in a horizontal section of the central disk) showing immunostained cells and processes beneath the folded coelomic epithelium and dense immunostaining in the basiepithelial nerve plexus beneath the epithelial lining of the eosophagus lumen. Abbreviations: BNP, basiepithelial nerve plexus; CE, coelomic epithelium; CS, cardiac stomach; CT, collagenous tissue; Es, esophagus; Mo, mouth; PM, peristomial membrane. Scale bars: 20 μm in (a), (c), (e); 100 μm in (b); 50 μm in (d)

Immunostained cells are present in the coelomic epithelium of the peristomial membrane and esophagus and in the underlying nerve plexus (Figure 15). The basiepithelial nerve plexus underlying the external epithelial layer of the peristomial membrane and esophagus also contains immunostained processes. Thus, the peristomial membrane and esophagus have two distinct layers of immunostained processes.

**Figure 16:**
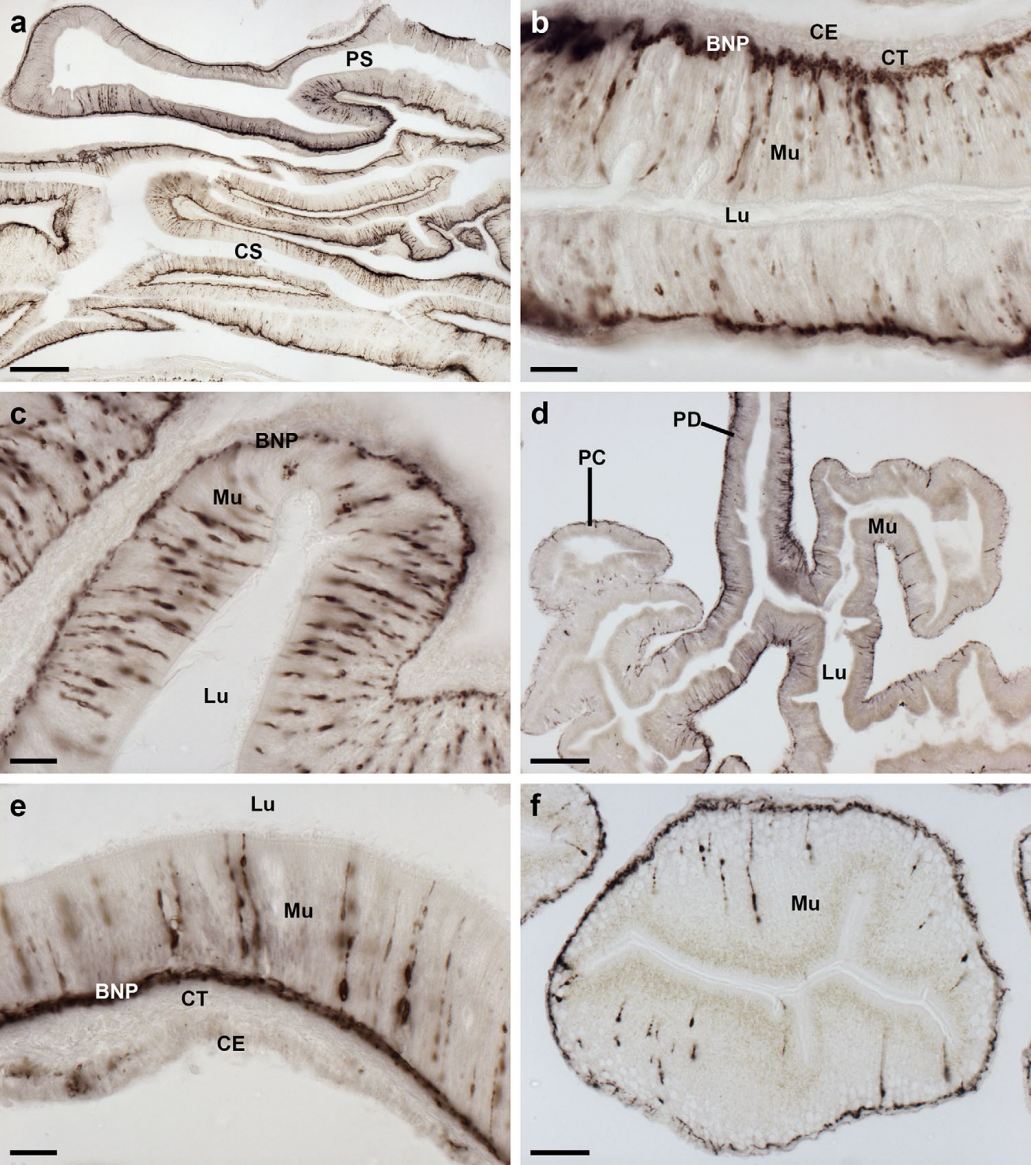
Localization of ArPPLN1b immunoreactivity in the cardiac stomach, pyloric stomach, pyloric duct and pyloric caeca of *A. rubens*. (a) Transverse section of the central disk region showing immunostaining in the cardiac stomach and pyloric stomach. (b) High magnification image of the cardiac stomach showing immunostaining in bipolar‐shaped cells in the mucosa and in the underlying basiepithelial nerve plexus. (c) High magnification image of the pyloric stomach showing immunostaining in bipolar‐shaped cells in the mucosa and in the underlying basiepithelial nerve plexus. (d) Horizontal section of the arm of a juvenile starfish showing immunostaining in the pyloric duct and pyloric caeca. (e) High magnification image of a transverse section of pyloric duct showing immunostaining in bipolar shaped mucosal cells and in the underlying basiepithelial nerve plexus. (f) High magnification image of a transverse section through a lobe of a pyloric caecum showing immunostaining in bipolar shaped mucosal cells and in the underlying basiepithelial nerve plexus (arrowheads). Abbreviations: BNP, basiepithelial nerve plexus; CE, coelomic epithelium; CS, cardiac stomach; CT, collagenous tissue; Lu, lumen; Mu, mucosa; PC, pyloric caeca; PD, pyloric duct; PS, pyloric stomach. Scale bars: 200 μm in (a); 20 μm in (b), (c), (d); 100 μm in (e); 50 μm in (f)

Immunostaining is present throughout the cardiac stomach (Figure 16a), including the floor of the cardiac stomach, the highly folded pouches of the cardiac stomach and the roof of the cardiac stomach. This immunostaining is localized in bipolar shaped cells present in the mucosal layer of the cardiac stomach and in their underlying processes which project into the intensely stained basiepithelial nerve plexus (Figure 16a, b). A similar pattern of immunostaining is present in the pyloric stomach (Figure 16a, c) and in the pyloric ducts (Figure 16d, e) that connect the pyloric stomach with the paired digestive glands (pyloric caeca) located in each arm (Figure 16d, f). Immunostained mucosal cells and processes in the underlying basiepithelial nerve plexus are also present in the pyloric caeca (Figure 16d, f).

#### 3.5.5 Coelomic epithelium, apical muscle, papulae and spines

**Figure 17:**
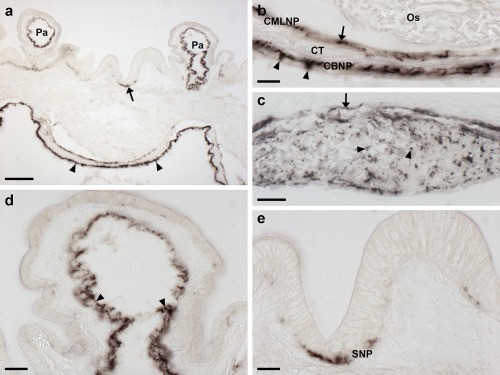
Localization of ArPPLN1b immunoreactivity in the body wall, apical muscle and body wall appendages of *A. rubens*. (a) Transverse section of aboral body wall showing immunostaining in the coelomic lining of the body wall (arrowheads), the coelomic lining of papulae and in the sub‐epithelial plexus (arrow) of the external body wall epithelium. (b) High magnification image of the coelomic lining of the body wall showing stained cells in the coelomic epithelium (arrowheads) and in the underlying basiepithelial nerve plexus. Immunostaining is also present in a nerve plexus (arrow) that is closely associated with a layer of circularly orientated muscle. (c) Immunostained axon profiles (arrowheads) in a transverse section of the apical muscle. Immunostained processes (arrow) can also be seen associated with a layer of circularly orientated muscle. (d) High magnification view of a longitudinal section of a papula showing immunostaining in the coelomic lining (arrowheads). (e) High magnification of the body wall external epithelium showing immunostaining in the sub‐epithelial nerve plexus. Abbreviations: CBNP, coelomic basiepithelial nerve plexus; CMLNP, circular muscle layer nerve plexus; CT, collagenous tissue; Os, ossicle; Pa, papulae; SNP, sub‐epithelial nerve plexus. Scale bars: 100 μm in (a); 20 μm in (b), (d), (e); 50 μm in (c)

Immunostained cells are present in the coelomic epithelium that forms the aboral lining of the coelom in each arm (Figure 17a, b). The processes of these cells form a sub‐epithelial plexus that is closely associated with an underlying thin layer of longitudinally orientated muscle (Figure 17c). Separated from the longitudinal muscle by a layer of collagenous tissue, immunostained processes are also present in close association with a layer of circularly orientated muscle. Thus, the coelomic lining is characterized by two separate layers of immunostained fibers. The apical muscle is a thickening of the longitudinal muscle layer of the coelomic lining, which runs in a sagittal position along the length of the arm into the central disk region. Immunostained cells are present in the coelomic epithelial lining of the apical muscle and the cross‐sectional profiles of immunostained processes can be seen within the apical muscle. As in other regions of the aboral coelomic lining, stained processes are also present in the thin circular muscle layer underneath the apical muscle. The pattern of immunostaining observed in the coelomic epithelium also extends into the lining of the papulae (Figure 17a, d), thin‐walled finger‐like structures that project externally through the body wall. Furthermore, immunostained processes are present in the sub‐epithelial plexus of the outer epithelium of the body wall (Figure 17a, e).

#### 3.5.6 Interossicular muscles

**Figure 18:**
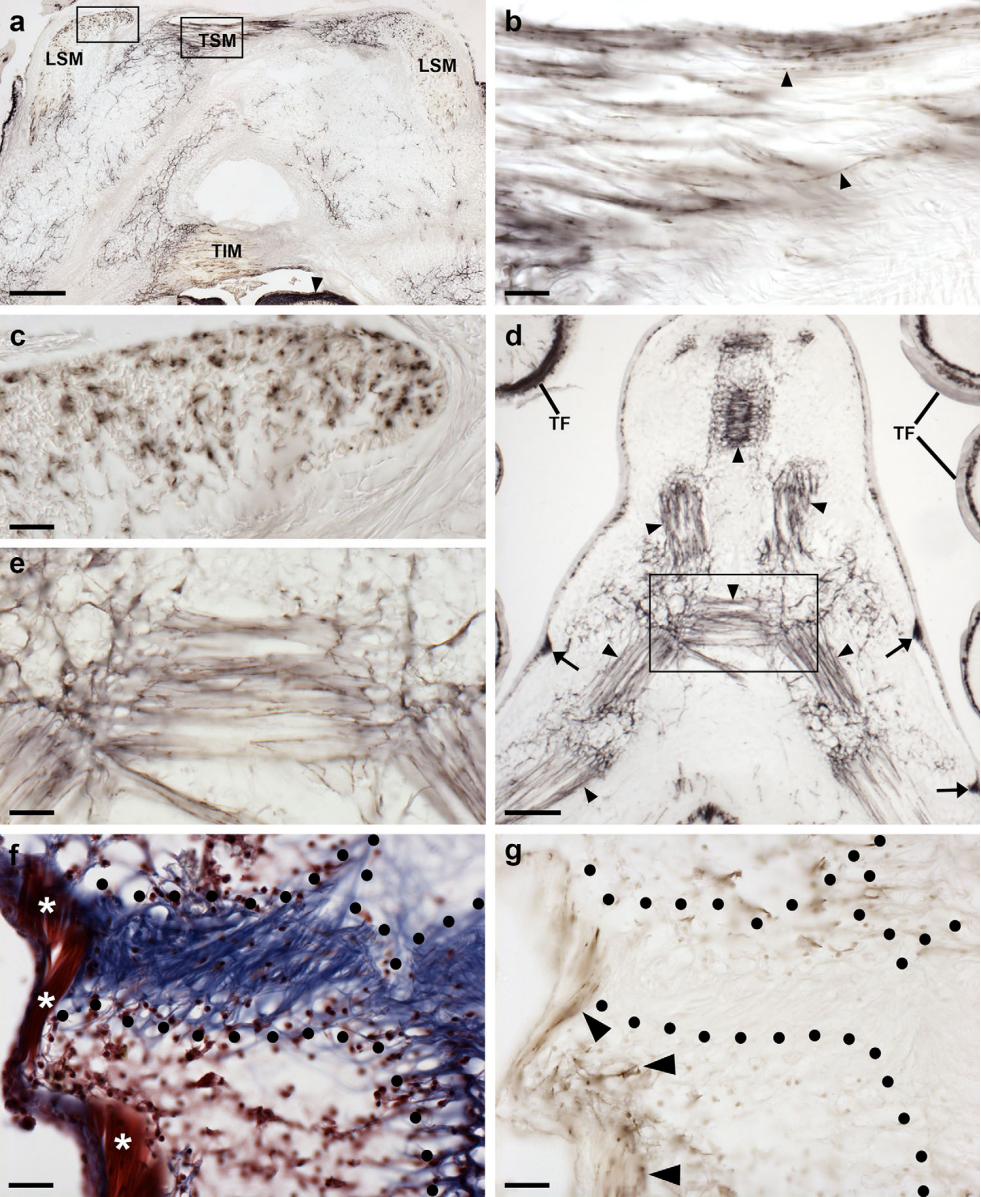
Localization of ArPPLN1b immunoreactivity in the innervation of interossicular muscles in *A. rubens*. (a) Transverse section of the ambulacrum showing immunostaining associated with muscles linking the ambulacral ossicles, which include the TSM, TIM, and LSM. The intensely stained radial nerve cord (arrowhead) can also be seen in this image. The boxed regions are shown at higher magnification in panels (b) and (c). (b) Immunostained nerve fibers (arrowheads) in a longitudinal section through the TSM. (c) Immunostained profiles of nerve fibers in a transverse section through the LSM. (d, e) Immunostaining in the body wall at the junction between two arms in a juvenile starfish. Immunostained fibers can be seen associated with muscles that link adambulacral ossicles (arrowheads). Stained fibers are also evident in thickenings of the sub‐epithelial nerve plexus of the body wall (arrows) and in the tube feet. A high magnification image of the boxed region is shown in (e). (f) Trichrome stained section of the body wall showing an interossicular muscle (white asterisks) and collagenous tissue (area bounded by black dots) linking adjacent ossicles. (g) Immunostained section adjacent to the section shown in panel (f). By comparing the immunostaining with the trichrome staining it can be seen that the immunostained fibers are associated with the interossicular muscle but not with the collagenous tissue (area bounded by black dots). Abbreviations: LSM, longitudinal supra‐ambulacral muscle; TF, tube foot; TIM, transverse infra‐ambulacral muscle; TSM, transverse supra‐ambulacral muscle. Scale bars: 200 μm in (a); 20 μm in (b), (c), (e), (f), (g); 100 μm in (d)

Immunostained nerve processes can be seen ramifying amongst the muscle fibers of interossicular muscles throughout the body wall (Figure 18a–e, g). Comparison of the immunostained sections with adjacent sections stained using Masson's trichrome method confirmed that the immunostained fibers are clearly associated with muscles linking adjacent skeletal osscicles and do not appear to be present in the adjacent interossicular collagenous ligaments (Figure 18f, g). Furthermore, the immunostained processes extend along the length of muscle fibers into the ossicles (Figure 18e, g).

### 3.6 ArPPLN1b causes dose‐dependent relaxation of in vitro preparations of apical muscle, tube feet and cardiac stomach from *A. rubens*

**Figure 19:**
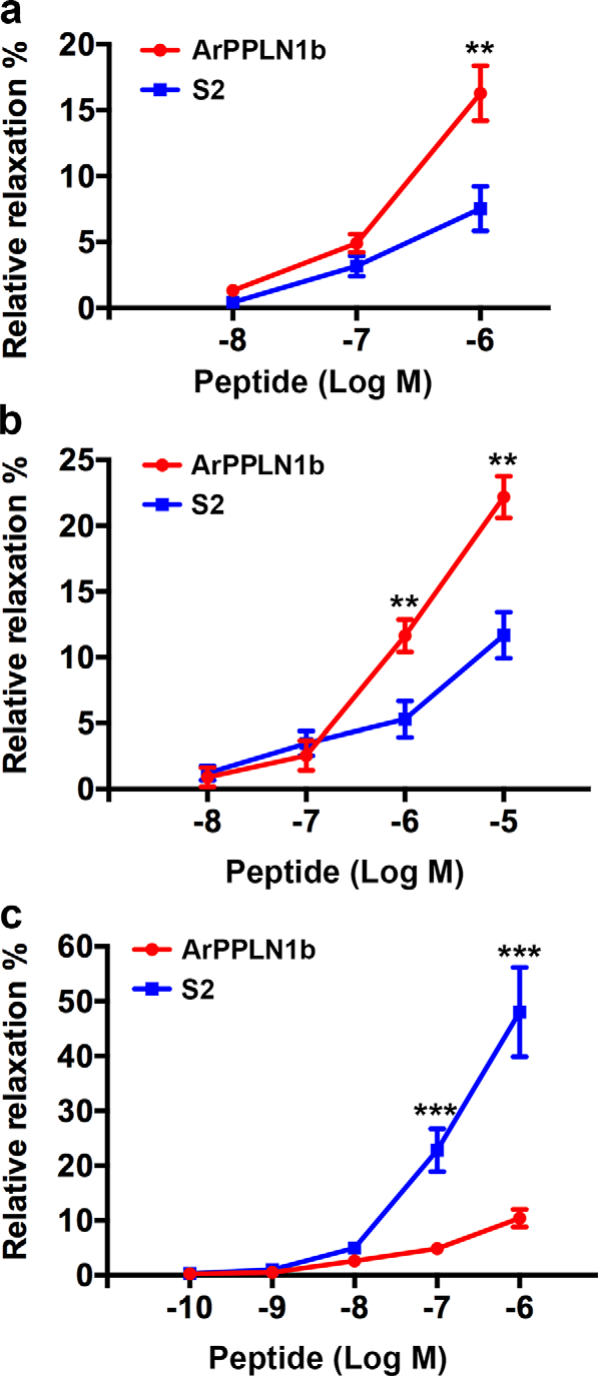
Graphs comparing the concentration‐dependent relaxing effects of ArPPLN1b and the SALMFamide neuropeptide S2 on in vitro apical muscle, tube foot and cardiac stomach preparations from *A. rubens*. Each point represents the mean ± *SEM* from at least four separate experiments, with the effect calculated as the percentage reversal of contraction induced by application of 10 µM acetylcholine (apical muscle and tube foot) or 30 mM KCl (stomach). (a) Apical muscle, where ArPPLN1b is more effective as a relaxant than S2 when tested at 10^−6^ M (*p* = .004; *t*‐test). (b) Tube foot, where ArPPLN1b is more effective as a relaxant than S2 when tested at 10^−6^ M (*p* = .007; *t*‐test) and 10^−5^ M (*p* = .001; *t*‐test). (c) Cardiac stomach, where ArPPLN1b is less effective as a relaxant than S2 when tested at 10^−7^ M (*p* = .0002; *t*‐test) and 10^−6^ M (*p* = .00002; *t*‐test)

ArPPLN1b caused dose‐dependent relaxation of apical muscle when tested at in vitro concentrations ranging from 10^−8^ to 10^−6^ M and was more effective than S2 as a relaxant of this preparation (Figure 19a). Thus, the relative relaxing effect of ArPPLN1b at 10^−6^ M was 16.29 ± 2.09% (*n* = 13), whereas the relative relaxing effect of S2 at 10^−6^ M was only 7.53 ± 1.70% (*n* = 11), which is only marginally higher than the relative relaxing effect ArPPLN1b at 10^−7^ M (4.90 ± 0.70%; *n* = 11).

ArPPLN1b caused dose‐dependent relaxation of tube feet when tested at in vitro concentrations ranging from 10^−8^ to 10^−5^ M and was more effective than S2 as a relaxant of this preparation (Figure 19b). Thus, the relative relaxing effect of ArPPLN1b at 10^−5^ M was 22.17 ± 1.59% (*n* = 6), whereas the relative relaxing effect of S2 at 10^−5^ M was only 11.67 ± 1.75% (*n* = 7), which is the same as the relative relaxing effect ArPPLN1b at 10^−6^ M (11.64 ± 1.24%; *n* = 6).

ArPPLN1b caused dose‐dependent relaxation of cardiac stomach when tested at in vitro concentrations ranging from 10^−10^ to 10^−6^ M but was much less effective than S2 as a relaxant of this preparation (Figure 19c). Thus, the relative relaxing effect of ArPPLN1b at 10^−6^ M was only 10.43 ± 1.60% (*n* = 11), whereas the relative relaxing effect of S2 at 10^−6^ M was 48.03 ± 8.17% (*n* = 5).

Control tests in which 20 µL of the vehicle (water) were added to the organ bath did not cause relaxation or contraction of any of the three preparations used here.

### 3.7 ArPPLN1b does not induce cardiac stomach eversion in *A. rubens*

Cardiac stomach eversion was observed within 5 min after injection of S2 in seven out of ten starfish. By comparison, stomach eversion was not observed in any of the ten starfish injected with ArPPLN1b, even after a 15 min period of observation.

## 4 DISCUSSION

The anatomical distribution and pharmacological actions of PP/OK‐type neuropeptides have been characterized in protostomian invertebrates, including mollusks and arthropods (Beck et al., 2000; De Lange, van Golen, & van Minnen, 1997; Dircksen et al., 2000; Hall & Lloyd, 1990; Jiang et al., 2015; Lloyd et al., 1996; Pearson & Lloyd, 1989; Wulff et al., 2017). Here we report the first detailed anatomical analysis of PP/OK‐type neuropeptide expression in a deuterostomian invertebrate, the starfish *A. rubens*. Use of both mRNA in situ hybridization and immunohistochemistry revealed a widespread pattern of expression of ArPPLNP1 and neuropeptides derived from this precursor, including cells and/or fibers in the radial nerve cords and circumoral nerve ring, tube feet, terminal tentacle, digestive system, coelomic lining, papulae and interossicular muscles. Consistent with previous studies on the starfish *P. pectinifera* (Kim et al., 2016) in vitro pharmacological tests revealed that the most abundant of the neuropeptides derived from ArPPLNP1 (ArPPLN1b or ArSMP) causes relaxation of apical muscle, tube foot and cardiac stomach preparations from *A. rubens*. Importantly, these findings indicate that PP/OK‐type neuropeptides may act as inhibitory neuromuscular transmitters in starfish. This is interesting because, as discussed in more detail below, it provides new insights into the mechanisms of neuromuscular signaling in echinoderms and the evolution of PP/OK‐type neuropeptide function in the animal kingdom. However, it would be simplistic to conclude that the sole function of PP/OK‐type neuropeptides derived from the ArPPLNP1 precursor is to act as inhibitory neuromuscular transmitters because the patterns of ArPPLN1b‐ir in *A. rubens* indicate signaling roles that extend beyond neuromuscular systems, as also discussed in more detail below.

### 4.1 Physiological interpretation of the anatomy and pharmacological actions of ArPPLNP1‐derived neuropeptides in *A. rubens*

#### 4.1.1 Radial nerve cords, circumoral nerve ring, tube feet and terminal tentacle

Localization of ArPPLNP1 expression in the radial nerve cords and circumoral nerve ring is consistent with the detection of ArPPLNP1 transcripts in the radial nerve cords of *A. rubens* and *P. pectinifera* using PCR (Kim et al., 2016). More specifically, the visualization of ArPPLNP1 transcripts in cells of both the ectoneural and hyponeural regions of the radial nerve cords and circumoral nerve ring can be interpreted from a physiological perspective. Our knowledge of the functional architecture of the nervous system of starfish and other echinoderms is clearly rather limited by comparison with other invertebrate species that are used as model systems in neurobiology (e.g., *Drosophila*, *C. elegans*, *Aplysia*). Nevertheless, anatomical and electrophysiological studies indicate that the hyponeural region has a purely motor function, whereas the ectoneural region comprises a mixed population of sensory neurons, interneurons, and motor neurons (Cobb, 1970; Mashanov, Zueva, Rubilar, Epherra, & Garcia‐Arraras, 2016; Smith, 1937, 1950). The primary function of the radial nerve cords and circumoral nerve ring is to co‐ordinate the activity of tube feet to enable whole‐animal locomotor activity (Smith, 1950). Therefore, the expression of ArPPLNP1 and neuropeptides derived from it in ectoneural cell bodies and their processes is indicative of roles in mediating intercellular signaling associated with neural control of tube foot‐mediated locomotor activity in starfish. The neuropile of the ectoneural region of the radial nerve cords and circumoral nerve ring is continuous with the basiepithelial nerve plexus of the tube feet. Therefore, ectoneural neurons in the radial nerve cords and circumoral nerve ring can, in principle, directly control tube foot activity. However, cells expressing ArPPLNP1 are also located along the length of the tube foot stem in *A. rubens* in close association with the basiepithelial nerve plexus, which is strongly immunoreactive with antibodies to ArPPLN1b. Accordingly, we show here that synthetic ArPPLN1b causes relaxation of *A. rubens* tube foot preparations in vitro. Thus, combining anatomical and pharmacological findings, neuropeptides derived from ArPPLNP1 may act physiologically to cause relaxation of the longitudinally orientated muscle layer in tube feet. This action could be functionally relevant for tube foot extension associated with the cyclical patterns of tube foot activity during starfish locomotor activity (Hennebert, Jangoux, & Flammang, 2013; Smith, 1950). Interestingly, ArPPLNP1 is also expressed by cells closely associated with the basal nerve ring in the tube foot sucker, which attaches to and detaches from the substratum during locomotor activity (Hennebert et al., 2013; Smith, 1950). ArPPLN1b‐ir nerve processes project into the tube foot sucker from the basal nerve ring, indicating a role in neural control of the sucker, and one possibility is that ArPPLNP1‐derived peptides are involved in regulation of the secretion of adhesive proteins (Hennebert, Wattiez, Waite, & Flammang, 2012).

The expression of ArPPLNP1 by cells in the hyponeural region of the radial nerve cords and circumoral nerve ring is consistent with the notion that neuropeptides derived from this precursor act as neuromuscular transmitters in starfish (Cobb, 1970; Mashanov et al., 2016; Smith, 1950). Thus, hyponeural neurons directly innervate the muscle layer of the ampullae, bulb‐shaped and fluid‐filled contractile organs that control the entry or withdrawal of fluid from the lumen of the tube feet during locomotor activity (Hennebert et al., 2013; Smith, 1950). In accordance with hyponeural expression of ArPPLNP1, ArPPLN1b‐ir nerve fibers are present in the ampulla (Figure 13e, f). If ArPPLNP1‐derived neuropeptides cause relaxation of muscle in the ampulla, then this action could be physiologically relevant in enabling accommodation of fluid in the ampulla during tube foot retraction. Furthermore, the axons of hyponeural neurons project to the lateral motor nerves (Smith, 1950), which also exhibit strong ArPPLN1b‐ir. This is the first study to demonstrate the presence of a neuropeptide in the lateral motor nerves, which is important because the presence of immunostained fibers in the lateral motor nerves is consistent with notion that ArPPLN1‐type neuropeptides act as neuromuscular transmitters that control the activity of body wall interossicular muscles, as discussed in more detail below.

The terminal tentacle is a specialized tube foot‐like sensory organ located at the tips of each of the arms in starfish, which can be extended and retracted (Hennebert et al., 2013; Lin et al., 2017). Consistent with the expression of ArPPLNP1 by cells along the length of tube foot stems, this precursor and neuropeptides derived from it are also expressed by cells in the wall of the terminal tentacle. Accordingly, by analogy with tube feet, neuropeptides derived from ArPPLNP1 could act as muscle relaxants to enable extension of the terminal tentacle. Interestingly, ArPPLNP1 expressing cells and ArPPLN1b‐ir cells and fibers are also present in flaps of tissue known as lateral lappets (Smith, 1937), which are located on both sides of the terminal tentacle. To the best of our knowledge, the functions of these structures have not been investigated. One possibility is that the lateral lappets have a chemosensory function, providing an expanded surface area for detection of environmental chemical cues from, for example, conspecifics or prey. It is noteworthy that ArPPLN1b‐ir is also present in the optic cushion, a photosensory organ located at the base of each terminal tentacle, and further studies are required to investigate the functional significance of this.

#### 4.1.2 Digestive system

ArPPLNP1transcripts and ArPPLN1b‐ir are present in many regions of the digestive system, including the peristomial membrane that surrounds the oral opening, the esophagus, cardiac stomach, pyloric stomach and the pyloric caeca. In vitro pharmacological tests with ArPPLN1b revealed that this peptide causes relaxation of cardiac stomach preparations. However, ArPPLN1b was less effective as a relaxant of the cardiac stomach than the *A. rubens* neuropeptide SALMFamide‐2 (S2). This contrasts with a previous study on the starfish *P. pectinifera*, where SMP (a homolog of ArPPLN1b) was found to be equally as effective as S2 in causing in vitro relaxation of the cardiac stomach (Kim et al., 2016). A likely explanation for this is that S2 is not a naturally occurring neuropeptide in this species, based on analysis of genome/transcriptome sequence data from the closely related species *P. miniata* (Elphick et al., 2015). In *P. miniata* there is an S2‐like peptide but its sequence is different to that of S2 from *A. rubens* and therefore S2 may be less effective as a cardiac stomach relaxant in *P. pectinifer*a than the naturally occurring S2‐like peptide in this species.

S2 belongs to a family of neuropeptides known as SALMFamides, which were the first echinoderm neuropeptides to be sequenced (Elphick, 2014). Immunocytochemical analysis of the expression of S2 in *A. rubens* reveals that, like ArPPLNP1‐derived neuropeptides, it is widely expressed, including the cardiac stomach and other regions of the digestive system (Newman et al., 1995a). Injection of *A. rubens* with S2 can induce cardiac stomach eversion, indicating a physiological role in the regulation of the extraoral feeding behavior of starfish (Melarange et al., 1999). Here we compared the effects of injection of equimolar amounts of S2 and ArPPLN1b in *A. rubens* and found that S2 induced stomach eversion in seven out ten animals but stomach eversion was not observed in any of the animals injected with ArPPLN1b. These findings are consistent with in vitro pharmacological experiments, where ArPPLN1b was found to be less effective as a cardiac stomach relaxant than S2. Collectively, these observations lead us to conclude that the physiological roles of ArPPLNP1‐derived neuropeptides in the digestive system *A. rubens* may not be limited to acting as muscle relaxants. If this is the case, then what other roles might ArPPLNP1‐derived neuropeptides have in the digestive system of *A. rubens*?

Previous studies have revealed that molluscan PPs stimulate ciliary activity on the surface of the foot in *Aplysia* and in the salivary ducts of *Tritonia* (Gaston, 1998; Willows et al., 1997). It is noteworthy, therefore, that cells expressing ArPPLNP1 and associated ArPPLN1b‐ir are located in regions of the digestive system in *A. rubens* that enable ciliary‐mediated movement of food material. For example, the pyloric ducts transfer food material from the pyloric stomach to the pyloric caeca. Experimental observation of ciliary‐generated currents has revealed that cells on the oral side of the pyloric duct generate centrifugal currents that move material from the pyloric stomach to pyloric caeca, whereas cells on the aboral side of the pyloric duct generate centripetal currents that move material from the pyloric caeca to the pyloric stomach (Irving, 1924; Jangoux, 1982). Interestingly, ArPPLNP1‐expressing cells (Figure 7a) appear to be more abundant on the oral side of the pyloric duct in *A. rubens*. Furthermore, ciliary‐mediated movement of food material occurs throughout the starfish digestive system (Anderson, 1953, 1954) and a potential role for ArPPLNP1‐derived neuropeptides is in regulation of ciliary‐mediated movement of food material through the digestive system of *A. rubens*.

#### 4.1.3 Coelomic lining, apical muscle, papulae and body wall sub‐epithelial plexus

The most prominent components of the nervous system in starfish are the ectoneural and hyponeural systems that form the radial nerve cords and circumoral nerve ring, as highlighted above. However, in crinoid echinoderms (e.g., feather stars) there is a third component to the nervous system that is more prominent than the ectoneural and hyponeural systems—this is the entoneural or aboral system (Mashanov et al., 2016). In starfish, the entoneural system is also present but is relatively inconspicuous when compared to that in crinoids. It comprises neurons located within the coelomic lining of the aboral body wall in close association with an underlying layer of longitudinally orientated muscle and another population of neurons associated with a deeper layer of circularly orientated muscle. A prominent feature associated with the aboral nervous system in starfish is the apical muscle, a thickening of the longitudinally orientated muscle layer that is located along the midline of each arm. Furthermore, from a functional perspective, contraction of the apical muscle facilitates flexion of the arms during behaviors that require changes in arm posture (e.g., feeding and righting behavior) (Smith, 1950).

It was the use of the apical muscle as a bioassay system for myoactive neuropeptides that enabled the purification of starfish myorelaxant peptide (SMP) from *P. pectinifera* (Kim et al., 2016). ArPPLN1b is a homolog of SMP from *P. pectinifera* and accordingly here we show that ArPPLN1b causes relaxation of in vitro preparations of the apical muscle from *A. rubens* (Figure 19a). Previous studies have reported that the SALMFamide neuropeptide S2 also causes relaxation of the apical muscle in *A. rubens*, although only at relatively high concentrations (Melarange & Elphick, 2003). Consistent with previously reported findings from *P. pectinifera*, here we show that ArPPLN1b is more potent/effective than the SALMFamide neuropeptide S2 in causing relaxation of the apical muscle. This contrasts with converse findings from cardiac stomach preparations, where S2 is more potent/effective than ArPPLN1b (see above and Figure 19c).

Consistent with the in vitro pharmacological effects of ArPPLN1b on apical muscle preparations, cells expressing ArPPLNP1 transcripts are located in the coelomic epithelium overlying the apical muscle (Figure 8a–d) and the immunostained axonal processes of these cells can be seen in transverse sections of the apical muscle (Figure 17c). Expression of ArPPLNP1 also extends beyond the apical muscle to cells in the coelomic epithelium located throughout the lining of the aboral body wall and accordingly ArPPLNP1‐ir is present throughout the longitudinally orientated muscle layer of the coelomic lining aborally. Immunostained processes are also present in the underlying circularly orientated muscle layer of the coelomic lining (Figure 17a), which may be derived from the lateral motor nerves (Smith, 1950).

The coelomic lining of the aboral body wall extends into papulae, thin‐walled and retractable dermal appendages that penetrate through the body wall to enable gas exchange between the external seawater and the coelomic fluid (Cobb, 1978). ArPPLN1b‐ir is present in fibers located within the coelomic lining of the papulae and these fibers are continuous with the innervation of the longitudinally and circularly orientated muscle layers of the coelomic lining of the aboral body wall (Figure 17a). Given that ArPPLN1b (ArSMP) acts as a relaxant of the apical muscle, it seems likely that ArPPLNP1‐derived neuropeptides also cause relaxation of muscle associated with the dermal papulae. Therefore, PP/OK‐type neuropeptides may participate in neural mechanisms controlling the protraction of papulae from the aboral body surface of starfish.

ArPPLN1b‐ir is also present within the sub‐epithelial nerve plexus below the external epithelium of the body wall (Figure 17a, e) and therefore ArPPLNP1‐derived peptides may also regulate the activity of other body wall associated appendages such as spines and pedicellariae.

#### 4.1.4 Interossicular muscles

One of the most striking features of ArPPLN1b‐ir in *A. rubens* is the presence of immunoreactive nerve fibers associated with interossicular muscles. The starfish endoskeleton comprises calcite ossicles that are interlinked by both collagenous ligaments and interossicular muscles (Blowes et al., 2017) and changes in body posture are mediated by changes in the contractile state of the muscles that connect adjacent ossicles. Importantly, this is the first study to report the presence of neuropeptide‐expressing nerve fibers in the interossicular muscles of starfish. Consistent with the presence of ArPPLN1b‐ir in nerve processes in the interossicular muscles, ArPPLN1b‐ir is also present in the lateral motor nerves (see above).

As ArPPLN1b (ArSMP) causes relaxation of muscle associated with the apical muscle, tube feet and cardiac stomach, it is likely that ArPPLNP1‐derived neuropeptides also act to cause relaxation of the interossicular muscles.

### 4.2 Comparative physiology of PP/OK‐type neuropeptide signaling in echinoderms

The discovery of a PP/OK‐type neuropeptide (SMP) that acts as a muscle relaxant in the starfish *P. pectinifera* provided the first insight into the physiological roles of PP/OK‐type neuropeptides in a deuterostome (Kim et al., 2016). Here characterization of a homolog of SMP in the starfish *A. rubens* (ArPPLN1b) has provided the first insights into the anatomy of PP/OK‐type neuropeptide signaling in a deuterostome. Collectively, the data obtained from both species indicate that SMP/PPLN1‐type neuropeptides have a general and widespread role as inhibitory neuromuscular transmitters in starfish.

Interestingly, PPLNP1 is not the only precursor of PP/OK‐type neuropeptides in starfish. Analysis of radial nerve cord transcriptome sequence data from *A. rubens* has revealed a partial sequence of a second precursor of PP/OK‐type neuropeptides, which was previously referred to as ArPPLNP (Semmens et al., 2016) but which we now refer to as ArPPLNP2. On‐going studies are directed toward determination of the complete sequence of ArPPLNP2, leading to identification and functional characterization of neuropeptides derived from this precursor. Looking ahead, it will be interesting to compare the physiological roles of neuropeptides derived from ArPPLNP1 and ArPPLNP2 in *A. rubens*.

From a developmental perspective, we recently reported localization of the expression several neuropeptide precursors in the larvae of *A. rubens*, using mRNA in situ hybridization (Mayorova et al., 2016). However, at the time this study was completed probes/antibodies for localization of the expression of ArPPLN1‐type neuropeptides were not available. With the generation of the ArPPLN1‐specific probes/antibodies reported here, there now exists an opportunity to extend analysis of expression of ArPPLN1‐type neuropeptides to the larval stages of *A. rubens*.

Another interesting avenue for future research on PP/OK‐type neuropeptides will be to compare the neuroanatomy of these signaling systems in different types of starfish, sampling species based on phylogenetic relationships. Such comparisons could reveal whether or not PPLN1‐type neuropeptides act as mediators of inhibitory neuromuscular transmission in all extant starfish species. Furthermore, comparison of species according to differences in ecological adaptations (e.g., burrowing versus non‐burrowing species; Santos, Haesaerts, Jangoux, & Flammang, 2005) might reveal how the physiology and anatomy of a neuropeptide signaling system in starfish has been adapted for life in different niches.

Extending the comparative approach further, precursors of PP/OK‐type neuropeptides have been identified in three of the four other extant echinoderm classes: echinoids (e.g., the sea urchin *S. purpuratus*; Rowe & Elphick, 2012), holothurians (e.g., the sea cucumber *A. japonicus*; Rowe, Achhala, & Elphick, 2014) and ophiuroids (e.g., the brittle star *Ophionotus victoriae*; Zandawala et al., 2017). However, nothing is known about the physiological roles of PP/OK‐type neuropeptides in other echinoderms. For example, it would be interesting to determine if PP/OK‐type neuropeptides act as inhibitory neuromuscular transmitters in all echinoderms.

Discovery of the effects of ArPPLN1b/SMP in causing muscle relaxation in starfish has provided the first insight into the physiological roles of PP/OK‐type neuropeptides in echinoderms, but it would be simplistic to conclude that the sole function of these neuropeptides in starfish is to act as inhibitory neuromuscular transmitters. For example, the abundance of ArPPLN1b‐ir in the radial nerve cords and circumoral nerve ring are indicative of roles in communication between neurons within these major components of the starfish nervous system. Furthermore, as discussed above, the presence of ArPPLN1b‐ir in regions of the digestive system involved in ciliary‐mediated transit of food material may be indicative of roles in neural control of ciliary activity, which would be consistent with the effects that PP‐type peptides have in causing an increase in ciliary beat frequency in mollusks (Hall & Lloyd, 1990).

### 4.3 The evolution of PP/OK‐type neuropeptide signaling

PP/OK‐type neuropeptides were first discovered in protostomian invertebrates (mollusks, arthropods) and the identification PP/OK‐type neuropeptide precursors in echinoderms established that the evolution of PP/OK‐type neuropeptides can be traced back to the common ancestor of protostomes and deuterostomes (Rowe & Elphick, 2012). The functional characterization of PP/OK‐type neuropeptides in starfish has provided the first opportunity to compare the physiological roles of PP/OK‐type neuropeptides in protostomian and deuterostomian invertebrates, thereby gaining insights into the evolution of PP/OK‐type signaling. A striking difference in the actions of PP/OK‐type neuropeptides in starfish and in protostomian invertebrates is their effects on muscle—in starfish PPLN1b/SMP acts as a muscle relaxant (Kim et al., 2016) whereas in protostomes PP/OK‐type neuropeptides are myocontractants (Dircksen et al., 2000; Stangier et al., 1992). Clearly, as highlighted above, a wider analysis of PP/OK‐type neuropeptide function in echinoderms is required to generalize findings from starfish. Nevertheless, the data currently available suggest that with the divergence of protostomes and deuterostomes, PP/OK‐type neuropeptides may have acquired opposite roles to act as myostimulatory peptides in protostomes and myoinhibitory peptides in deuterostomes. However, testing the generality of this hypothesis may prove to be difficult because thus far PP/OK‐type neuropeptides have not been identified in the other deuterostomian phyla—the hemichordates and chordates, which may reflect loss of PP/OK‐type signaling in these phyla.

One of the challenges associated with investigation of the evolution of PP/OK‐type neuropeptide signaling is that receptors for this family of neuropeptides have as yet not been identified in any species. Investigation of the evolution of other neuropeptide signaling systems has revealed that in some cases the ligands for orthologous neuropeptide receptors can diverge to such an extent over large evolutionary timescales that they are not recognizable as orthologous neuropeptides based on sequence similarity. A good example of this would be neuropeptide‐S (NPS) in vertebrates and crustacean cardioactive peptide (CCAP)‐type neuropeptides in protostomes, which exhibit very little sequence similarity in spite of being ligands for orthologous receptors (Mirabeau & Joly, 2013; Semmens et al., 2015). Likewise, the possibility remains that orthologs of the protostome/echinoderm PP/OK‐type neuropeptides exist in hemichordates and/or chordates, but have not been identified thus far due to sequence divergence. For this reason, discovery of receptors for PP/OK‐type neuropeptides is an imperative for reconstruction of the evolution of this signaling system in the animal kingdom. Intriguingly, there is some evidence that the evolutionary origin of PP/OK‐type neuropeptide signaling may extend beyond the Bilateria to other metazoans because the SITFamide precursor in the placozoan *Trichoplax adhaerens* comprises multiple copies of peptides that share some sequence similarity with PP/OK‐type neuropeptides (Nikitin, 2015). This similarity may, of course, simply reflect convergence. Nevertheless, it highlights the need for further characterization of PP/OK‐type neuropeptide signaling in a range of phyla and discovery of the receptors that mediate the effects of these neuropeptides.

## ORCID

*Maurice R. Elphick* http://orcid.org/0000-0002-9169-0048
